# A COVID-19-association-dependent categorization of death causes in 100 autopsy cases

**DOI:** 10.1007/s11357-021-00451-w

**Published:** 2021-09-11

**Authors:** Krisztina Danics, Adrián Pesti, Klára Törő, Noémi Kiss-Dala, János Szlávik, Botond Lakatos, Andrea Radnai, Tamás Balázs, Miklós Bacskai, Deján Dobi, Tibor Várkonyi, Tibor Glasz, Gábor Lotz, András Kiss, Zsuzsa Schaff, István Vályi-Nagy

**Affiliations:** 1 Department of Forensic and Insurance Medicine, Semmelweis University, Budapest, Hungary; 2 2nd Department of Pathology, Semmelweis University, Budapest, Hungary; 3 Central Hospital of Southern Pest-National Institute of Hematology and Infectious Diseases, Budapest, Hungary; 4 Heathware Consulting Ltd, Budapest, Hungary

**Keywords:** Autopsy, Cause of death, COVID-19, Histopathology, SARS-CoV-2

## Abstract

From March through December 2020, 100 autopsies were performed (Semmelweis University, Budapest, Hungary), with chart review, of patients with severe acute respiratory syndrome coronavirus 2 (SARS-CoV-2) infection demonstrated by real-time reverse-transcription polymerase chain reaction testing (mean age, 74.73 years, range 40–102 years; 50 males, mean age 71.96 years, and 50 females, mean age 77.5 years). Classified by the date of death, 21 cases were from the pandemic’s “first wave” (March through July) and 79 from the “second wave” (August through December). Three mortality categories were defined by relevance of SARS-CoV-2 infection: (1) “strong” association (*n*=57), in which COVID-19 was primary responsible for death; (2) “contributive” association (*n*=27), in which a pre-existing condition independent of COVID-19 was primary responsible for death, albeit with substantial COVID-19 co-morbidity; (3) “weak” association (*n*=16), in which COVID-19 was minimally or not at all responsible for death. Distributions among categories differed between the first wave, in which the “contributive” association cases dominated (strong: 24%, contributive: *48%*, weak: 28%), and the second wave, in which the “strong” association cases dominated (strong: *66%*, contributive: 21%, weak: 13%). Charted co-morbidities included hypertension (85 %), cardiovascular diseases (71 %), diabetes (40 %), cerebrovascular diseases (31 %), chronic respiratory diseases (30 %), malignant tumors (20 %), renal diseases (19 %), diseases of the central nervous system (15 %), and liver diseases (6 %). Autopsy evaluation analyzed alterations on macroscopy as well as findings on microscopy of scanned and scored sections of formalin-fixed, paraffin-embedded tissue samples (50–80 blocks/case). Severity of histological abnormalities in the lung differed significantly between “strong” and “contributive” (*p*<0.0001) and between “strong” and “weak” categories (*p*<0.0001). Abnormalities included diffuse alveolar damage, macrophage infiltration, and vascular and alveolar fibrin aggregates (lung), with macro- and microvascular thrombi and thromboemboli (lung, kidney, liver). In conclusion, autopsies clarified in what extent COVID-19 was responsible for death, demonstrated the pathological background of clinical signs and symptoms, and identified organ alterations that led to the death. Clinicopathologic correlation, with conference discussions of severity of co-morbidities and of direct pathological signs of disease, permitted accurate categorization of cause of death and COVID-19 association as “strong,” “contributive,” or “weak.” Lung involvement, with reduced ventilatory capacity, was the primary cause of death in the “strong” and “contributive” categories. Shifts in distribution among categories, with “strong” association between COVID-19 and death dominating in the second wave, may reflect improved clinical management of COVID-19 as expertise grew.

**Supplementary Information:**

The online version contains supplementary material available at 10.1007/s11357-021-00451-w.

## Introduction

Since the onset of the present pandemic of severe acute respiratory syndrome coronavirus 2 (SARS-CoV-2) infection [1], several studies have presented the results of autopsies performed in coronavirus disease 2019 (COVID-19) [2–22]. Systematic meta-analyses also have appeared [18, 23, 24]. These studies, however, are limited by smaller numbers within individual series, excepting two single-center reports, one with 80 cases [10] and another with 100 autopsies [7], and a multi-institutional report with 135 [12]. Observers initially described the COVID-19 as a respiratory disease; however, clinical and pathological analyses soon discovered rapid progression of more severe COVID-19 to multiple organ dysfunction syndrome [25, 26].

Autopsies in COVID-19 ideally should detail organ alterations at gross and microscopic levels, describe evidence of immunological reactions, weigh the significance of pre-existing co-morbidities, and correlate clinical and pathological data. Post-mortem studies are expected to explain the significance of factors involved in disease severity and to identify those leading to death. Histological analysis of tissues may help to clarify the pathogenic mechanism involved in COVID-19 and how the SARS-CoV-2 infection leads to more or less severe organ alterations and even to death. Despite various studies, however, answers to several questions are still not clear [27].

SARS-CoV-2 infection was identified in Hungary in March 2020 [28]. Autopsies were thenceforward performed regularly in COVID-19 patients who died in hospitals. We here present the results of 100 autopsies in patients with documented SARS-Cov-2 infection starting from March through December 2020. The autopsies were carried out in the Department of Forensic and Insurance Medicine, in collaboration with the 2^nd^ Department of Pathology of the Semmelweis University, Budapest, Hungary. The autopsied subjects died at the South-Pest Central Hospital and National Institute of Hematology and Infectious Diseases (DPCK), where the patients were diagnosed and treated using standard procedures.

## Material and methods

**Fig. 1:**
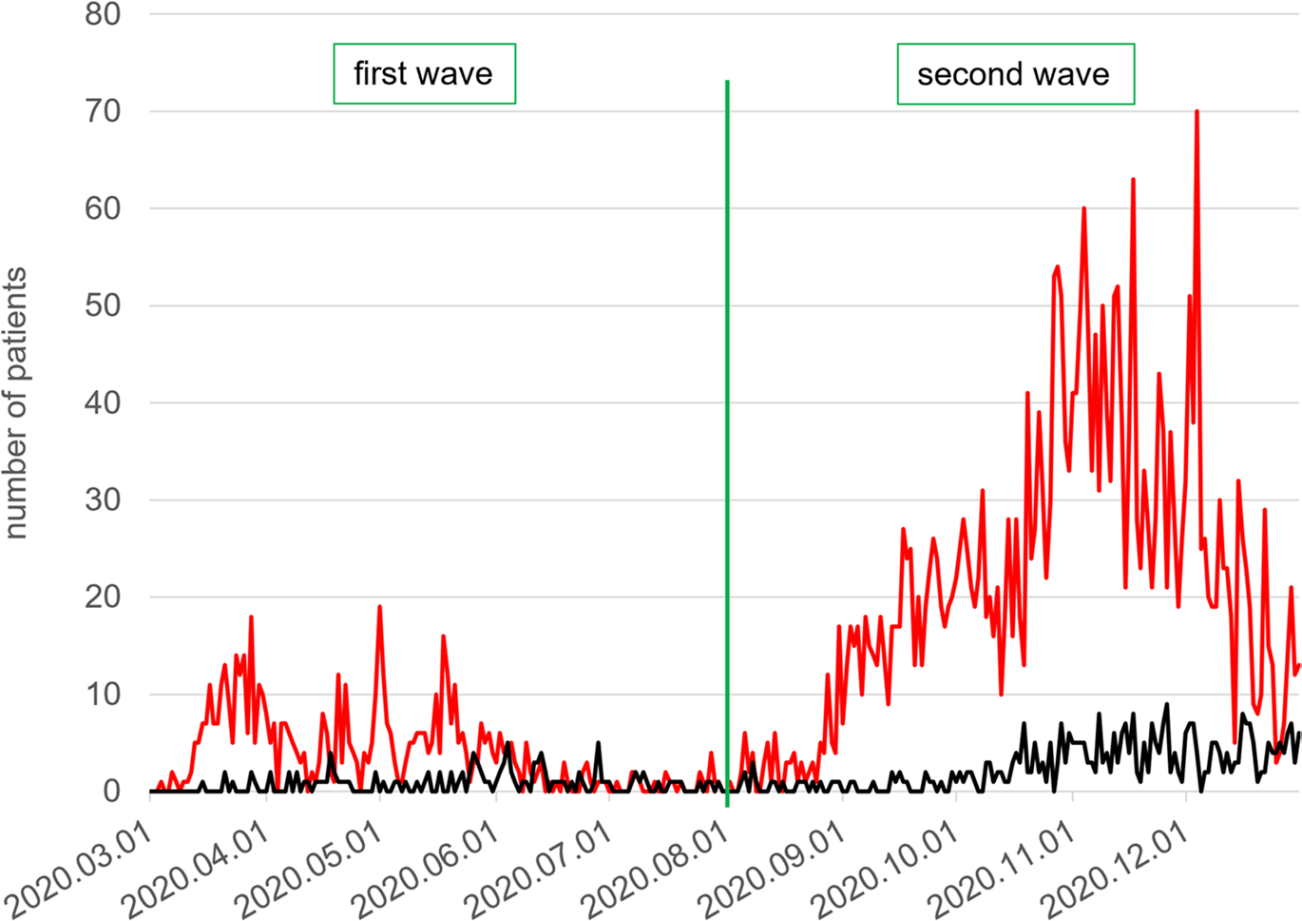
Patients, South-Pest Central Hospital and National Institute of Hematology and Infectious diseases (DPCK) and total number and deaths, with SARS-CoV-2 infection demonstrated by RT-PCR in the first wave (March–July) and second wave (August–December 2020) of the pandemic. Red line, all patients with the SARS-CoV-2 infection; black line, cohort of such patients who died.

From March through December 2020, real-time reverse-transcription polymerase chain reaction (RT-PCR) testing (performed at the Department of Laboratory Medicine of Semmelweis University) demonstrated SARS-CoV-2 sequences in 3814 patients visiting the out- or in-patient departments of DPCK. Among them, 495 died. These patients were identified through linking the National COVID-19 Registry files holding all results of SARS-CoV-2 RT-PCR testing and DPCK in- and out-patient records. The pandemic period was divided into a first wave (March through July 2020) and a second wave (August through December 2020) (Fig. 1).

Among the 495 dead DPCK patients, 100, randomly selected, underwent autopsy in the Department of Forensic and Insurance Medicine of the Semmelweis University. The same prosectors, using standard infectious-disease precautionary protocols, conducted all autopsies. Histopathological, immunohistochemical, and clinicopathological analyses were carried out in collaboration with the 2^nd^ Department of Pathology of Semmelweis University. The study was in accordance with the Declaration of Helsinki and was approved by the Central Medical Ethical Committee, Budapest, Hungary (No.IV/3961-2/2020/EKU, IV/4986-1/2020/EKU). The diagnosis of COVID-19 was based on results of ante-mortem RT-PCR testing, with documentation of SARS-CoV-2 sequences in oro- and nasopharyngeal fluid. Most patients had definitive or suspect signs or symptoms of COVID-19, including compatible imaging-study (radiographic) findings.

Demographic data (age, sex); co-morbidities such as hypertension, diabetes, malignant tumors, and cardiovascular, cerebrovascular, hepatobiliary, chronic respiratory-tracheal, renal, and central nervous system diseases; and body mass index (BMI) were noted (Table 1), as well as clinical-laboratory values (Table 2). Age at death was recorded. Patient’s co-morbidities, characteristics of hospitalizations (duration, intensive care, mechanical ventilation, etc.) were identified by interrogating National COVID-19 Registry data, DPCK in- and out-patient records, and National Health Insurance Fund (NHIF) administrative records. The NHIF database includes data on all patients’ state-funded services since 2000, with diagnoses recorded as ICD-10 codes (International Classification of Diseases, 10th Revision, Hungarian version), interventions as ICHI codes (International Classification of Health Interventions), and uses DRGs (Diagnosis-Related Groups) as classifiers of episodes of hospital admission. At least 2 entries of the same 5-digit ICD-10 code in a patient’s administrative records defined a diagnosis, and then these diagnoses were grouped into co-morbidity categories as described [29]. All documents concerning medical history, radiology, laboratory data, and treatments, together with death certificates, were recorded and provided to the prosection team before autopsies were performed. The post-mortem interval (PMI) to autopsies varied between <1 and 10 days (average 4.25 days; Suppl. Table 1). The prosector could start the autopsy once the cadaver and a detailed medical history were available, and this for most autopsies was with PMI between <1 and 4 days (58 autopsies).

**Table 1:** Demographic and clinical characteristics of autopsied patients with SARS-CoV-2 (*n*=100)

*n* (number of patients)	100
Females; *n* (%)	50 (50.0)
Age, years; mean (SD)	74.73 (13.11)
Age, females, years; mean (SD)	77.50 (13.08)
Age, males, years; mean (SD)	71.96 (12.67)
Age categories, years; *n* (%)	
<51	7 (7.0)
51–60	3 (3.0)
61–70	23 (23.0)
71–80	34 (34.0)
81–90	23 (23.0)
>90	10 (10.0)
Co-morbidities in patient history; *n* (%)	
Hypertension	85 (85.0)
Cardiovascular diseases	71 (71.0)
Diabetes	40 (40.0)
Cerebrovascular diseases	31 (31.0)
Respiratory diseases	30 (30.0)
Malignant tumors	20 (20.0)
Renal diseases	19 (19.0)
Diseases of the central nervous system	15 (15.0)
Liver diseases	6 (6.0)
BMI, kg/m^2^; mean (SD)	28.93 (7.20)
BMI categories; *n* (%)	
<18.5	1 (1.0)
18.5–24.9	29 (29.0)
25–29.9	34 (34.0)
>30	36 (36.0)
Length of hospital stay, days; mean (SD)	18.62 (22.03)
Length of hospital stay categories, days; *n* (%)	
<1	0 (0)
1–2	13 (13.0)
3–4	6 (6.0)
5–9	22 (22.0)
10–15	20 (20.0)
>15	39 (39.0)
Intensive care = yes; *n* (%)	63 (63.0)
Cause of death categories; *n* (%)	
Strong COVID-19	57 (57.0)
Contributive COVID-19	27 (27.0)
Weak COVID-19	16 (16.0)
Pandemic waves; *n* (%)	
First wave	21 (21.0)
Second wave	79 (79.0)

*SD* standard deviation, *BMI* body mass index

**Table 2:** Abnormal laboratory data categorized according to the cause of death with deviations from the normal range

	Deceased with available laboratory measurement *n*	Deceased with abnormal laboratory value *n* (%)	Cause of death categories with respect to type of COVID-19-association			*p*-value
			Strong *n* (%)	Contributive *n* (%)	Weak *n* (%)	
Total number of deceased			57	27	16	
Elevated alkaline phosphatase (%) Normal range: 40–130 U/l	82	28 (34.1)	17 (32.7)	5 (29.4)	6 (46.2)	0.599
Elevated bilirubin (%) Normal range: 5–21 µmol/l	81	15 (18.5)	10 (19.2)	0 (0.0)	5 (38.5)	0.029
Elevated CKMB (%) Normal range: < 24 U/l	58	27 (46.6)	15 (36.6)	7 (58.3)	5 (100.0)	0.018
Elevated CRP (%) Normal range: < 10 mg/l	89	84 (94.4)	51 (94.4)	18 (90.0)	15 (100.0)	0.445
Elevated D-Dimer (%) Normal range: < 500 ng/ml	84	80 (95.2)	50 (96.2)	16 (88.9)	14 (100.0)	0.445
Low eGFR (CKD-EPI) (%) Normal range: > 60 ml/min/1.73 m^2^	89	49 (55.1)	31 (57.4)	10 (50.0)	8 (53.3)	0.841
Elevated ferritin (%) Normal range: 15–300 ng/ml	80	69 (86.3)	44 (89.8)	14 (77.8)	11 (84.6)	0.441
Elevated GGT (%) Normal range: 12–52 U/l	82	63 (76.8)	44 (84.6)	12 (70.6)	7 (53.8)	0.05
Elevated AST (%) Normal range: 13–40 U/l	82	41 (50.0)	29 (55.8)	7 (41.2)	5 (38.5)	0.384
Elevated ALT (%) Normal range: 7–40 U/l	82	27 (32.9)	18 (34.6)	5 (29.4)	4 (30.8)	0.91
hs-Troponin I (%) Normal range: < 0.03 ng/ml	75	45 (60.0)	27 (55.1)	9 (60.0)	9 (81.8)	0.263
Interleukin-6 (%) Normal range: 0.64–10 pg/ml	75	69 (92.0)	46 (93.9)	13 (86.7)	10 (90.9)	0.66
Elevated LDH (%) Normal range: 240–480 U/l	85	63 (74.1)	44 (83.0)	10 (55.6)	9 (64.3)	0.047
Low lymphocyte (%) Normal range: 1.5–4 G/l	91	74 (81.3)	44 (81.5)	19 (86.4)	11 (73.3)	0.607
Elevated neutrophil (%) Normal range: 2–7.5 G/l	91	60 (65.9)	40 (74.1)	12 (54.5)	8 (53.3)	0.141
Elevated NT-proBNP (%) Normal range: 35–125 pg/ml	57	56 (98.2)	40 (97.6)	11 (100.0)	5 (100.0)	0.82
Elevated urea nitrogen (%) Normal range: 3–8 mmol/l	89	66 (74.2)	46 (85.2)	13 (65.0)	7 (46.7)	0.006
Elevated creatinine (%) Normal range: 61.9–114.9 µmol/l	89	40 (44.9)	25 (46.3)	9 (45.0)	6 (40.0)	0.91

*n* = number of decedents (% with elevated values in the category). *p*-value shows the difference among the categories (*p*<0.05)

*ALP* alkaline phosphatase, *CKMB* creatinine kinase myocardial band, *CRP* C-reactive protein, *eGFR (CKD-EPI)* estimated glomerular filtration rate from the Chronic Kidney Disease Epidemiology Collaboration equation, *GGT* gamma-glutamyltransferase, *hs-Troponin* high-sensitivity Troponin, *AST* aspartate aminotransferase, *ALT* alanine aminotransferase, *LDH* lactate dehydrogenase, *NT-proBNP* N-terminal prohormone of brain natriuretic peptide

Serum biochemistry values were retrieved from the DPCK’s information system and included alkaline phosphatase (ALP), serum bilirubin, creatinine kinase myocardial band (CKMB), C-reactive protein (CRP), D-Dimer, estimated glomerular filtration rate (eGFR CKD-EPI), ferritin, gamma-glutamyl transferase (GGT), aspartate aminotransferase (AST), alanine aminotransferase (ALT), high-sensitivity troponin T (hs-TNT), interleukin-6 (IL6), lactate dehydrogenase (LDH), lymphocyte count, neutrophil granulocyte count, N-terminal prohormone of brain natriuretic peptide (NT-proBNP), urea nitrogen, and creatinine. Median values of the serum biochemistries from the 7-day period before the decedents’ death were calculated and recorded as lower–normal/elevated for eGFR CKD-EPI and lymphocyte count and elevated–non-elevated for the rest of the parameters (Table 2).

Organ alterations identified on macroscopy were documented by photography. Tissue samples for histological examination (50–80 blocks/case) were taken. Samples were obtained from the lungs (each lobe, 3–4/lobe), trachea, bronchi, pulmonary-hilus lymph nodes, heart (left and right atria, ventricles), liver, both kidneys, oropharynx, thyroid glands, spleen, adrenal glands, bone marrow, gastrointestinal tract (stomach, small and large intestines), brain, and gonads, as well as to include sites of identified macroscopic alterations.

The tissue blocks were fixed in buffered 10% formalin with a final formaldehyde concentration of 4%, embedded in paraffin (FFPE). Sections picked up on glass slides were routinely stained with hematoxylin and eosin (HE) and periodic acid-Schiff techniques. All slides bearing HE-stained sections were scanned using a 3DHistech Pannoramic® 1000 Digital Slide Scanner (3D Histech Ltd, Budapest, Hungary) with 82× optical magnification (0.121 µm/pixel resolution). The resulting images were evaluated and scored by board-certified pathologists. To reduce interobserver variability, 2–3 pathologists, who were experts in lung and heart (TG, KD, TV), kidney (DD, GL), and liver (ZsS, GL) pathology independently examined and scored the scanned images during the first sitting. A second sitting was organized among the organ experts in case of disagreement and questionable, complicated cases were discussed by the whole group of pathologists participating in the study in a third sitting to reach final conclusion and scoring. Severity of the alterations was analyzed semiquantitatively, in the lung, heart, kidney, and liver as 0=none, 1=mild, 2=moderate, and 3=severe, as appropriate. A supplementary histologic evaluation round was implemented by summing up single pulmonary alteration term values on a case-by-case basis to yield case determined “total pulmonary histo-scores” in order to more precisely estimate the impact of pulmonary pathology on the mechanism of death.

### Immunohistochemistry

Immunohistochemical (IH) studies were conducted using sections of the blocks described above taken from the lungs, cut at 4 µm and picked up on coated glass slides. IH reactions were carried out in 25 cases, in which the PMI was <4 days, and additional 5–5 cases between 5–6, 7–8, and 9–10 days PMI using 2 blocks from each lung tissue sample. In case no IH reaction was identified, additional 3^rd^ blocks were used. To detect SARS-CoV-2 nucleocapsid protein, a mouse monoclonal antibody (IgG1, clone #05, Cat. No. 40143-MM05, Sino Biological Inc., Beijing, P.R. China) was used in 1:1000 dilution. To detect SARS-CoV-2 spike protein, a rabbit monoclonal antibody (IgG clone HL6; Cat. No. GTX635654, GeneTex, Inc., Irvine, CA, USA) was used in 1:250 dilution. Stainings were carried out in a Ventana Benchmark Ultra automated immunohistochemistry system (Ventana Medical Systems, Inc., Tucson, AZ, USA) using a staining protocol including steps of heat pre-treatment with pH=9 Cell Conditioning 1 and signal development with OptiView DAB IHC Detection Kit (both Ventana Medical System, diaminobenzidine chromogen) and hematoxylin counterstaining.

### Statistical analysis

All continuous data are presented as the mean ± standard deviation (SD) or median ± interquartile range (IQR), with categorical data presented as numbers and percentages. Fisher’s exact tests were used to analyze categorical variables. Age and BMI variables of the main groups were compared by ANOVA testing, while for the length of hospitalization variable, Kruskal-Wallis testing was applied. Normal distribution assumptions were analyzed by histograms. These statistical analyses were performed by R (version 4.0.2), using package tableone (version 0.12.0). *P*-value < 0.05 was considered statistically significant for all analyses.

## Results

### Demographic, clinical, and laboratory findings

From March to December 2020, 495 DPCK patients tested positive for SARS-CoV-2 and died; out of them, autopsies were done in 100 cases at the Department of Forensic and Insurance Medicine and analyzed by histology in collaboration with the 2^nd^ Department of Pathology of the Semmelweis University, Budapest, Hungary. The most important demographic and clinical data are listed in Table 1. There were 50 male and 50 female patients; the mean age of the decedents was 74.73 (SD 13.11) years ranging from 40 to 102. The mean age was significantly different by gender [mean age (SD), years; males 71.96 (12.67) and females 77.50 (13.08); *p*=0.034)]. There was also a difference in the mean age by pandemic waves, but it did not reach statistical significance [mean age (SD), years; “first wave” 77.90 (14.33) and “second wave” 73.89 (12.73); *p*=0.214].

The duration of hospitalization ranged from 1 to 116 days [mean (SD) 18.62 (22.03), median 13.0] (Table 1). There was a statistically significant difference between the two waves in the median length of hospitalization [median (IQR) days; “first wave” 24.00 (15.00, 38.00) and “second wave” 10.00 (5.00, 17.50); *p*<0.001)] (Suppl. Table 2). Sixty-three patients were admitted to the intensive care unit (ICU) (Table 1).

All but one SARS-CoV-2-infected deceased had at least one of the examined pre-existing diseases; 89 of them had more than one. The co-morbidities are the following in order of decreasing frequency: hypertension 85 %, cardiovascular diseases 71 %, diabetes 40 %, cerebrovascular diseases 31 %, chronic respiratory diseases 30 %, malignant tumors 20 %, renal diseases 19 %, diseases of the central nervous system 15 %, and liver diseases 6 %. Most of the decedents were either overweight (34) or obese (36) resulting in a 28.93 (7.2) kg/m^2^ [mean (SD)] BMI (Table 1).

### Serum biochemistry findings

The most important abnormalities in serum biochemistries, which were evaluated further in the different death categories (see later), are listed in Table 2.

CRP, D-Dimer, ferritin, and interleukin-6 elevation were the most commonly altered values in comparison to normal range. Percentages of deceased with elevated bilirubin, CKMB, LDH, and urea nitrogen values were significantly different according to our novel, categorized cause of death groups (see later). Elevated bilirubin and elevated CKMB were most common in weak associated with COVID-19 death category, while elevated LDH and urea nitrogen being the most common in strong associated with COVID-19 category (see later) (Table 2).

### Pathology findings

**Fig. 2:**
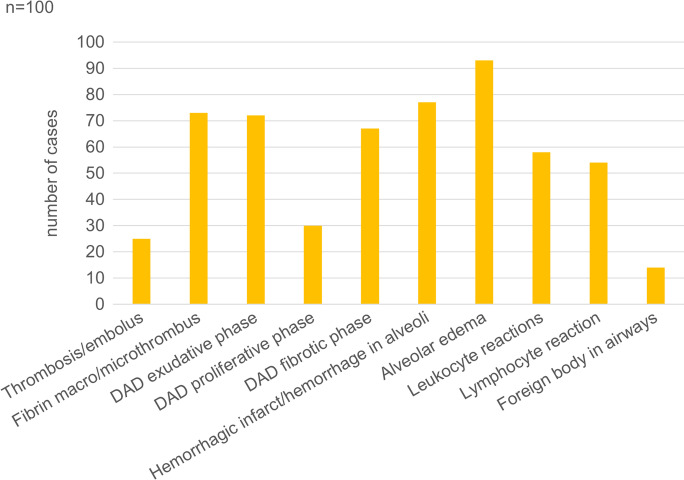
Percentage of patients with pulmonary alterations detected by histopathologic examination among 100 autopsied subjects with SARS-CoV-2 infection. DAD, diffuse alveolar damage.

**Fig. 3:**
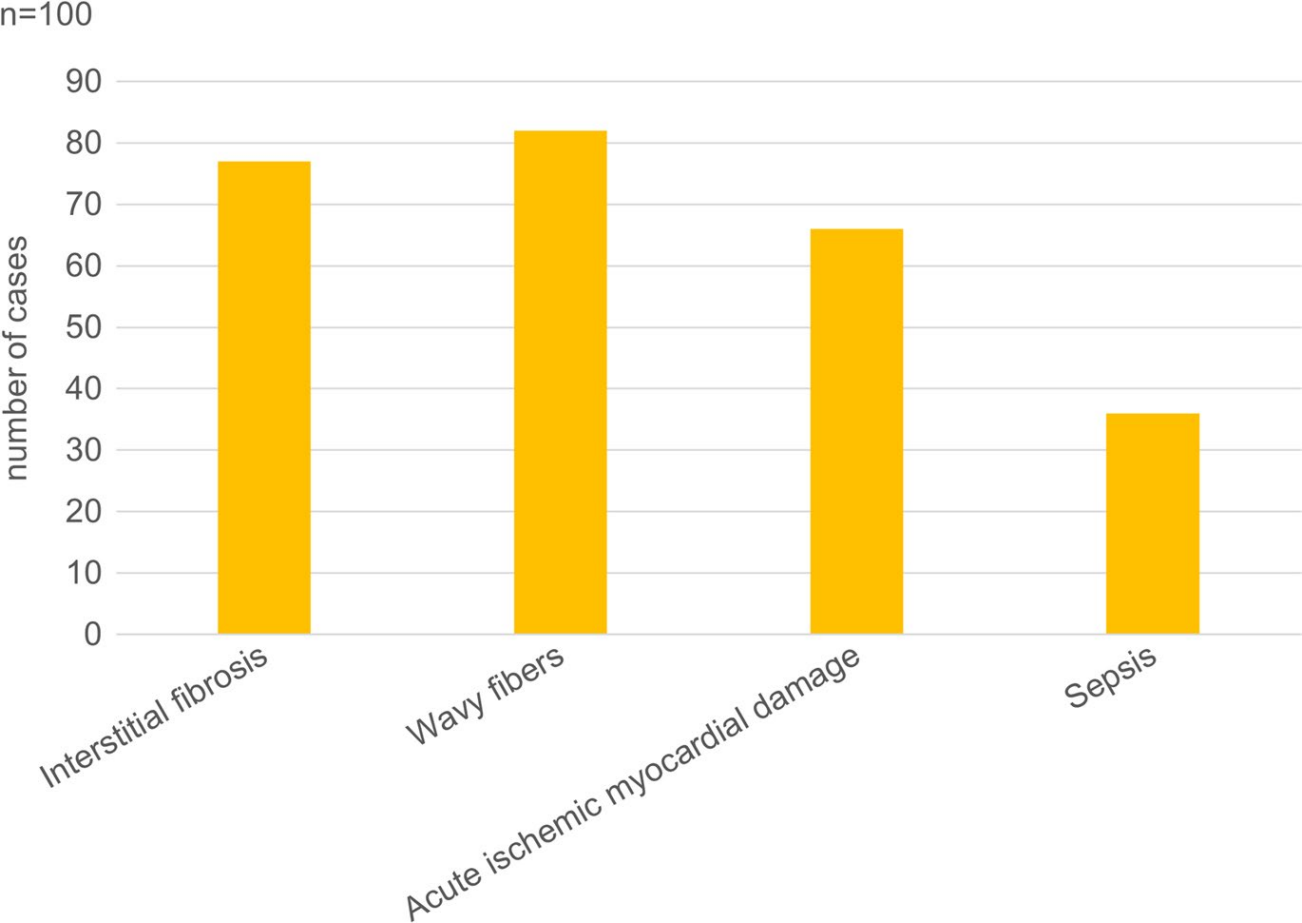
Percentage of patients with cardiac alterations detected by histopathologic examination among 100 autopsied subjects with SARS-CoV-2 infection.

**Fig. 4:**
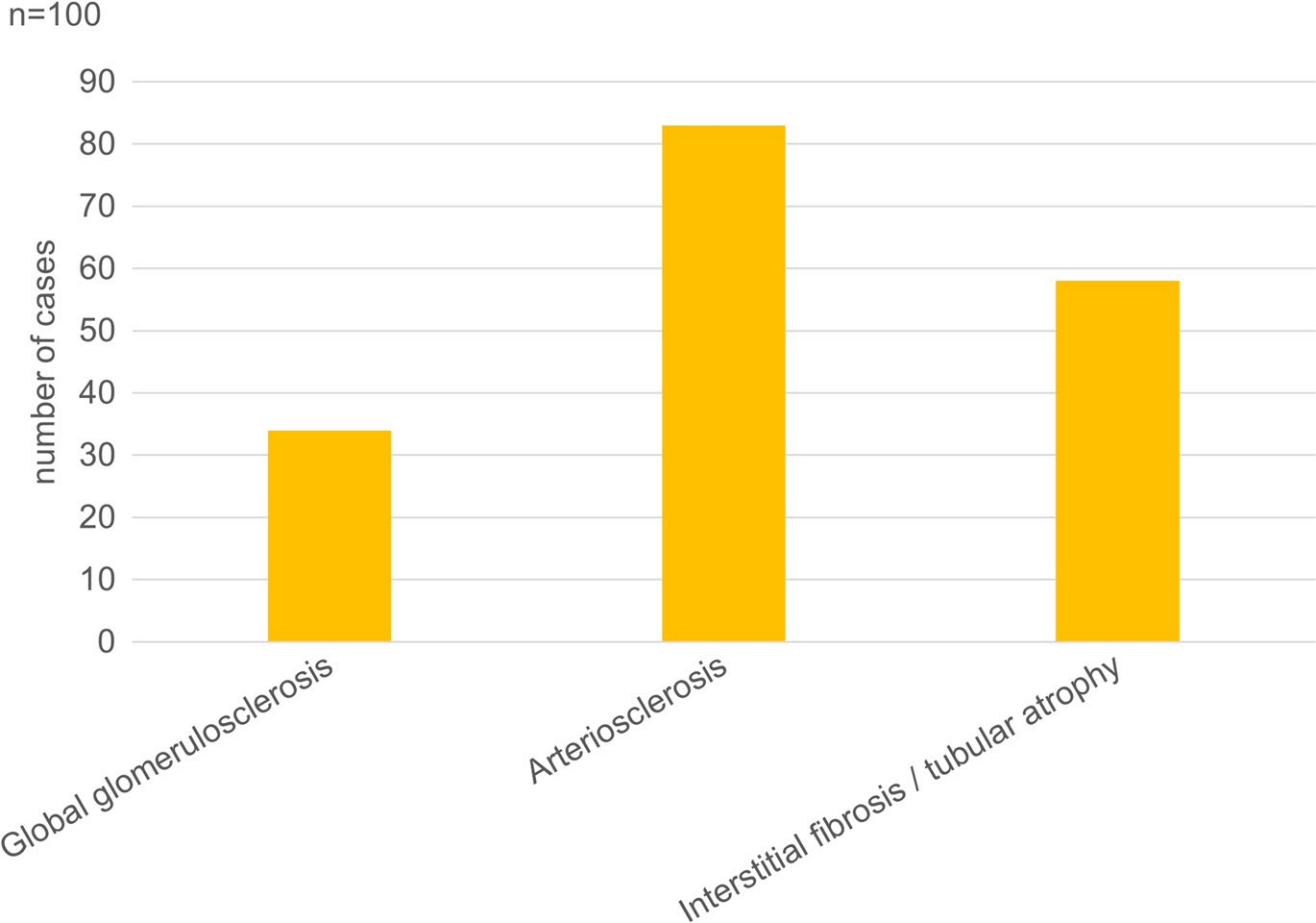
Percentage of patients with kidney alterations detected by histopathologic examination among 100 autopsied subjects with SARS-CoV-2 infection.

**Fig. 5:**
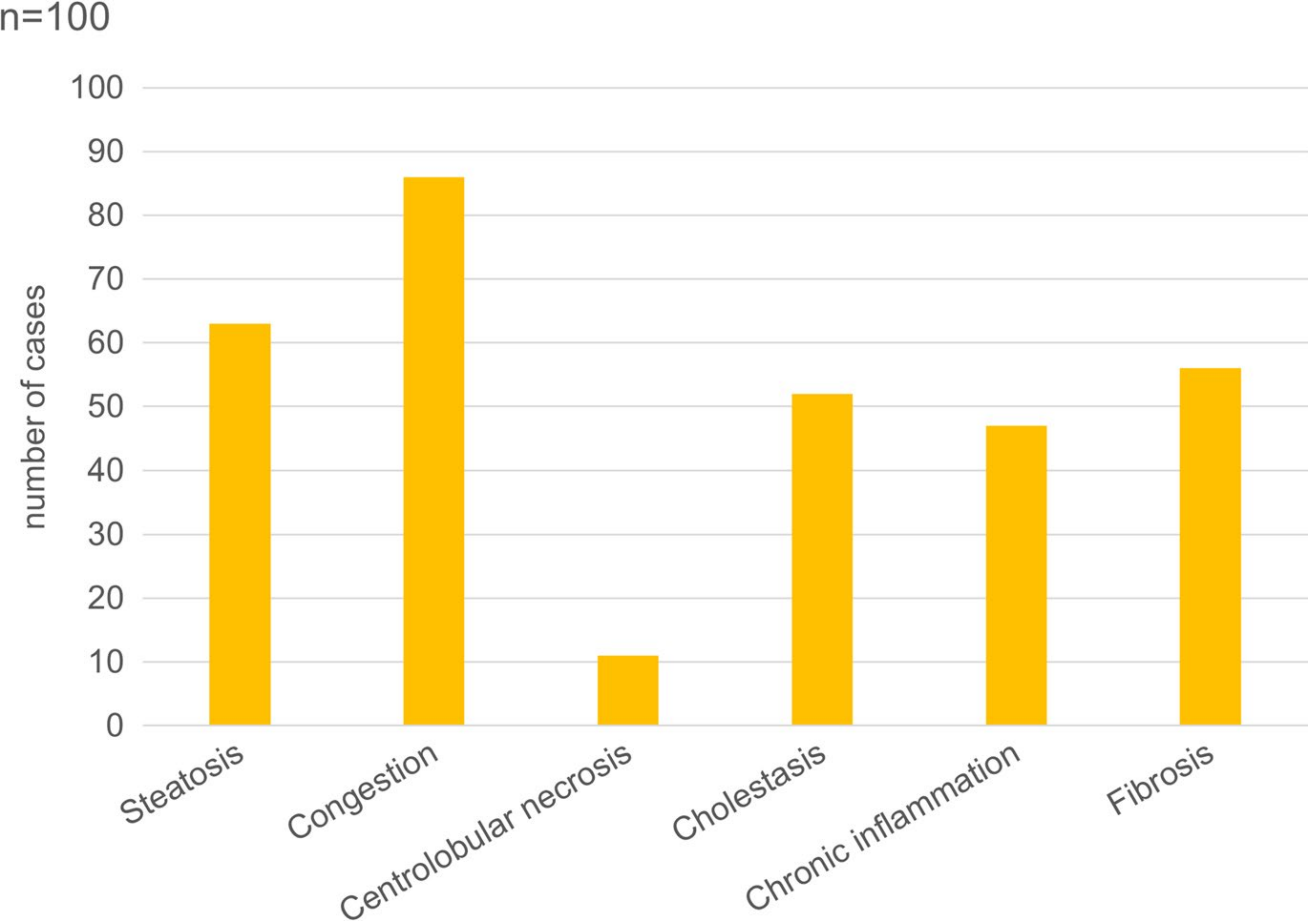
Percentage of patients with liver alterations detected by histopathologic examination among 100 autopsied subjects with SARS-CoV-2 infection.

The results of the semiquantitative histological evaluation (scoring) of the lung, heart, kidney, and liver in the decedents are presented in Figs. 2, 3, 4 and 5.

#### Lungs

**Fig. 6:**
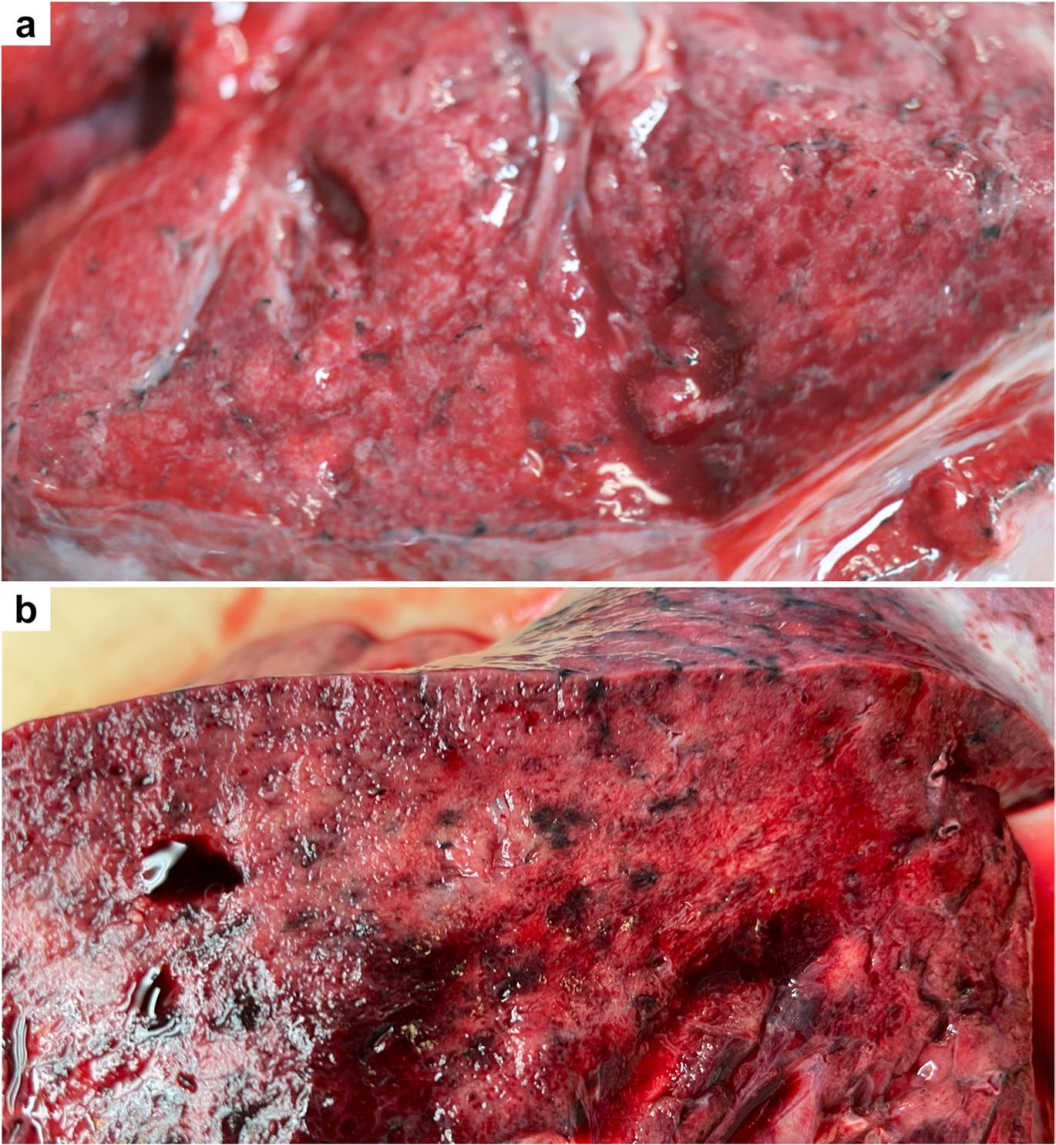
Gross pulmonary alterations in COVID-19. Massive consolidation with congested, airless, and edematous lungs with a fine lacelike dotted pattern on cut surface (**a**) and hemorrhages of varying extent (**b**).

**Fig. 7:**
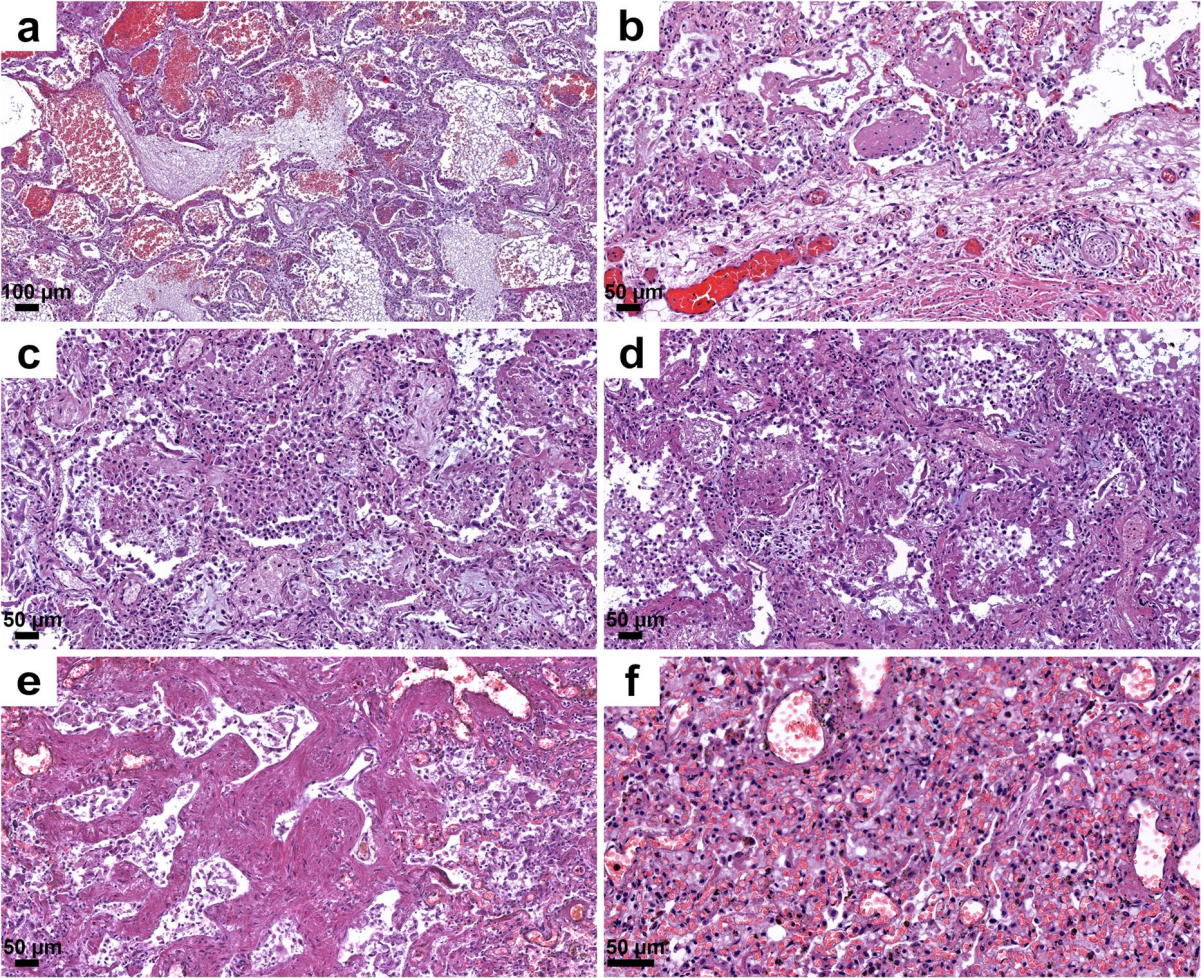
Histopathologic findings, lung, in COVID-19. Protein-rich edema fluid, intraalveolar hemorrhage (**a**), alveolar fibrin plugs and hyaline membranes (**b**), accumulation of desquamated and inflammatory cells (**c**), proliferation of fibroblasts (**d**), septal fibrosis corresponding to macroscopic honeycombing (**e**), and alveolar collapse (**f**). Hematoxylin/eosin (HE).

**Fig. 8:**
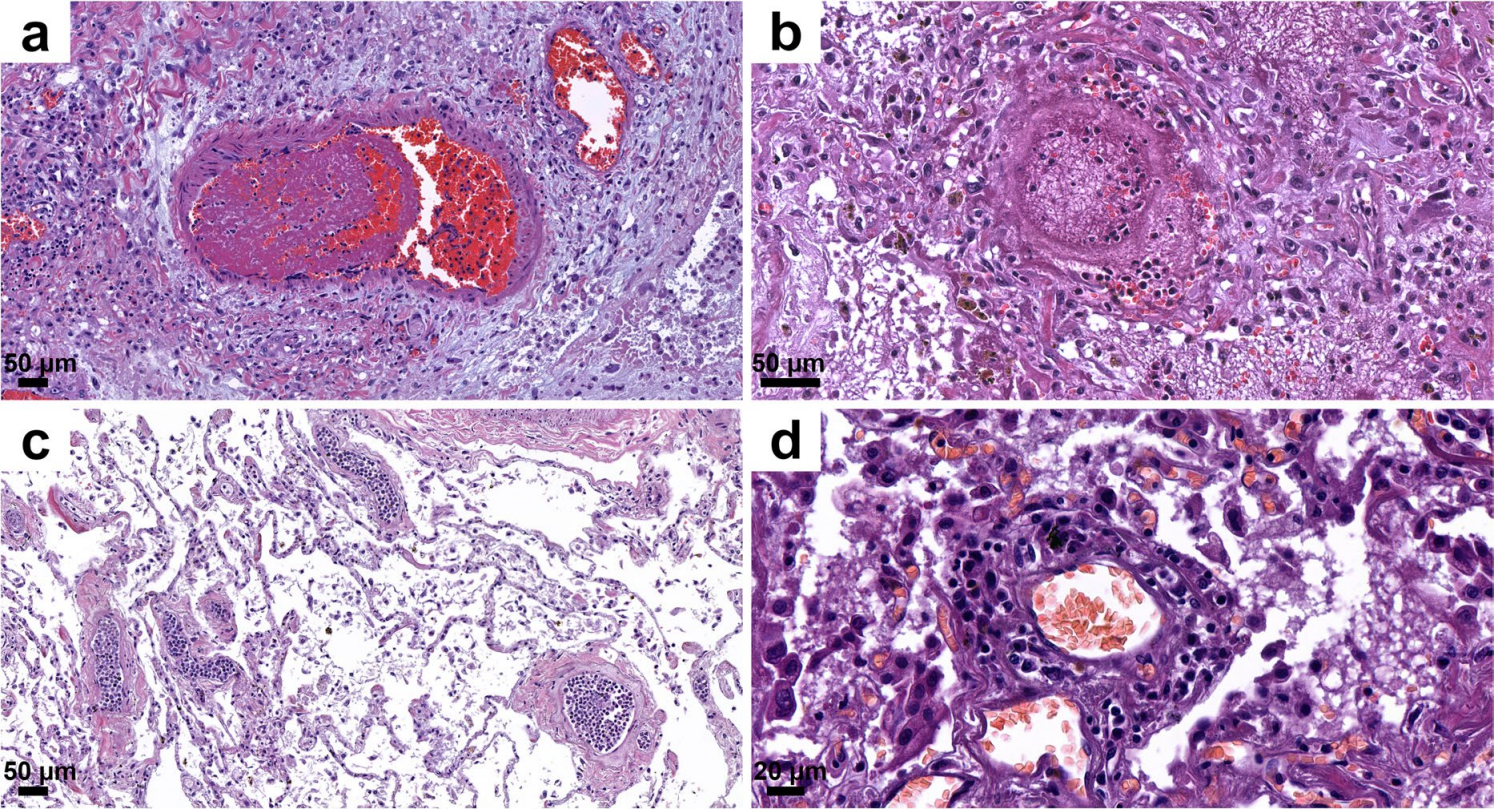
Histopathologic findings, lung, in COVID-19. Microthrombi and aggregates of mononuclear cells (“cellular-sludge”) in pulmonary vessels (**a**,**b**, **c**) and vasculitis (**d**). HE.

**Fig. 9:**
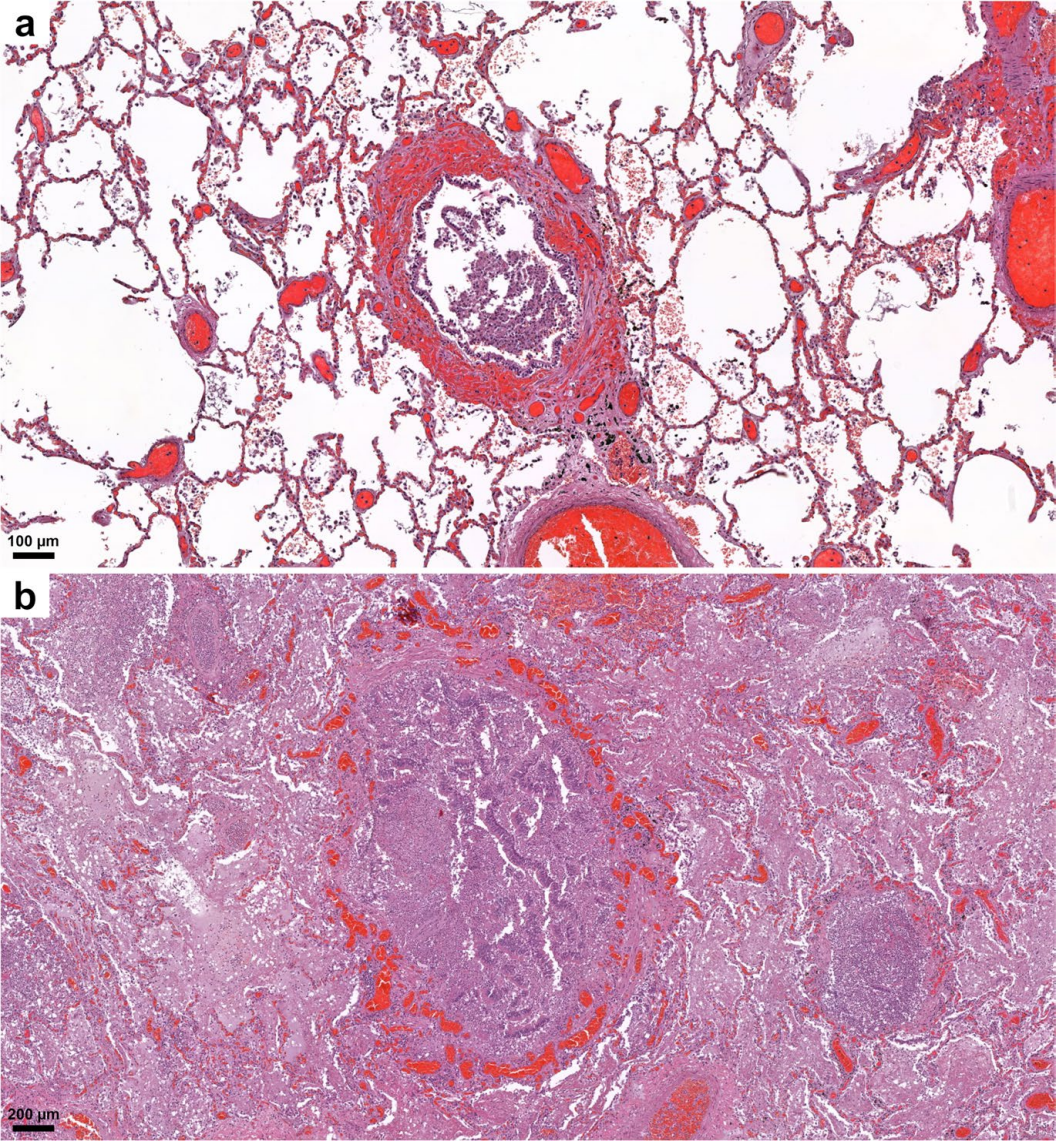
Histopathologic findings, lung, in COVID-19. Bronchopneumonia with many neutrophils and dilated, blood-filled capillaries cuffing bronchi in a “ring” or “corona”–like pattern (**a**, **b**). HE.

The lungs were overweight (left lung weight averaged 820 g, range: 282–1742 g, and right 965 g, range: 376–2270 g). In the majority of the cases, the lung parenchyma was consolidated, congested, and airless making the impression of raspberry jelly, exhibiting increased fragility under finger compression, typically yielding quantities of extractable edema with a fine lacelike dotted pattern on cut surfaces (Fig. 6a) and dark-colored hemorrhages of various extent (Fig. 6b). Emphysema of various degrees were detected in all lungs. Histologic picture was strongly dominated by an overall airlessness of the lung parenchyma due to alveolar edema and a multitude of alveolar appearances summarized under the generic term DAD (diffuse alveolar damage) (Figs. 2 and 7) This manifested within single cases in the kaleidoscope of thin-to-protein-rich edema fluid, hyaline membrane-type fibrin deposits mixed with debris of macrophages, alveolar cell remnants, alveolar fibrin plugs, air space blood extravasates, and accumulation of desquamated cell debris with macrophage elements (Fig. 7a, b, c). Such early changes represented exudative-type DAD and were in fact mostly of non-inflammatory nature, thus deserving the term “Covid-pneumopathy” rather than pneumonia as their apt designation. Short- and long-term secondary events such as focal-to-regional granulocyte accumulation corresponding to bacterial superinfective purulent bronchopneumonia as well as insudation of fibrin deposits into alveolar septa soliciting fibroblast proliferation (proliferation-type DAD) (Fig. 7d) and progression to septal fibrosis (fibrotic-type DAD) were noted (Fig. 7e), resulting in the later aspect of lung tissue honeycombing. Similarly, progression of alveolar fibrin plugs to fibrosis resulted diffuse fibrotic areas without honeycombing. Reactive cellular changes like pneumocyte hyperplasia, squamous metaplasia, and multinucleated giant cells were detected as well. Thrombotic changes of the pulmonary vasculature, both microthrombosis (<1 mm) and macrothrombosis (>1 mm) were seen, the first being more common (Fig. 8a). In some cases, microvessel lumina were stenosed by a meshwork of fibrin fibers or aggregates of mononuclear inflammatory cells (Fig. 8b, c). Occasionally, vasculitis was experienced (Fig. 8d) and dilated capillaries surrounded the inflammated bronchi in a “ring or corona-like pattern” (Fig. 9a, b). Bronchopneumonia with large number of neutrophils was detected occasionally as sign of bacterial superinfection (Fig. 9b). In summary, pulmonary findings dominated deaths in strong association with COVID-19 category on exhibiting a characteristic pattern of the above-quoted individual histologic changes. The percentage of cases with the individual histologic alterations is presented in Fig. 2.

### Extrapulmonary organ alterations

The alterations in other organs were generally less severe when compared with the lungs both by gross pathology and by histology. The changes were mainly associated with pre-existent chronic diseases or with vascular alterations.

#### Heart

**Fig. 10:**
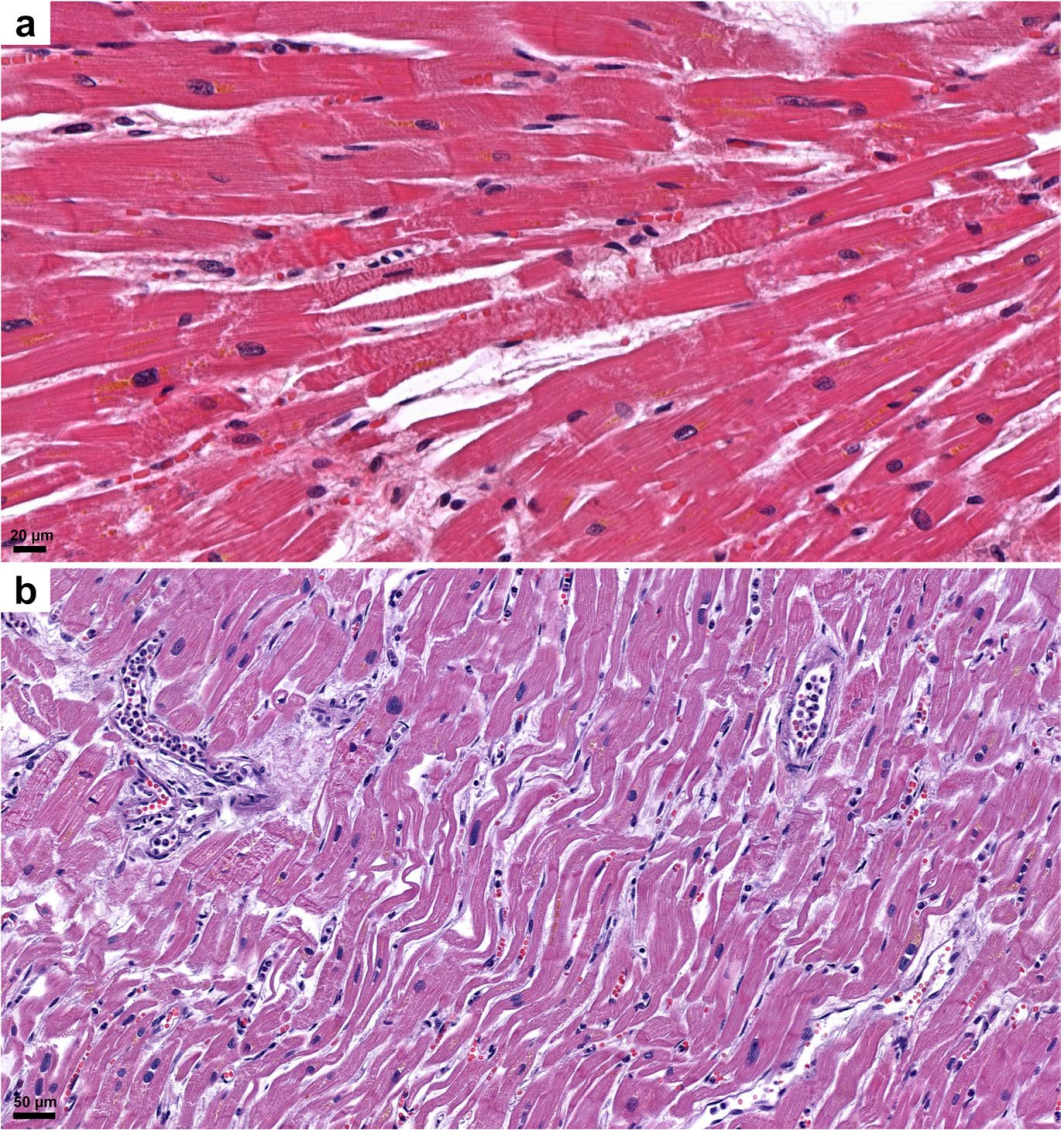
Histopathologic findings, heart, in COVID-19. “Patchy” hyperacute myocardial damage demonstrating ruptured fibers and contraction bands amidst striated viable muscle cells (**a**). Wavy myocardial fibers, extreme dilation of reserve capillaries, “patchy” myocardial damage, and cellular sludge within small vessels (**b**). HE.

Heart specimens demonstrated an almost critical average overweight (472 g, range 204–1030 g). Typically, cardiac cavities were flaccidly dilated with various degree of coronary artery disease. Extensive post-mortem tissue sampling of left and right heart myocardium as well as the coronary segments with most severe sclerosis demonstrated interstitial fibrosis of various degree in 77% (Figs. 3 and 10a). Chronic myocardial damage and vessel remodeling indicated histories of cardiovascular ischemic disease originating years before fatal SARS-CoV-2 infection. Their contributive significance to fatal outcome was appreciated via 2-scale (absent: 0; present: 1) and 4-scale (absent: 0; mild: 1; moderate: 2; severe: 3) semiquantitative evaluation scores as appropriate. There were, however, no significant differences in the chronic myocardial damage and coronary sclerosis among the three mortality categories (see latter). Acute myocardial damage was scored according to special extension: irregularly distributed acute myocyte damage in areas <1 cm in diameter was termed “patchy” acute myocardial damage, whereas the same in an area >1 cm in diameter was termed acute myocardial infarction (AMI). “Patchy” acute myocyte damage was found in 50% of the patients, AMI was present in 15%, and no acute type myocardial damage was detected in 35%. Another form of acute myocardial circulatory derangement was (non-extensive) fibrin microthrombosis in 8% of patients. Sepsis was indicated by a more-than-usual number of acute inflammatory cells within intramyocardial capillaries (27%) or as septic thrombi associated with the ventricular endocardium (18%) (Figs. 3 and 10b). Myocarditis was present in 3% of the cases. So called “wavy” fibers were present in 82% (Figs. 3 and 10b).

#### Kidneys

**Fig. 11:**
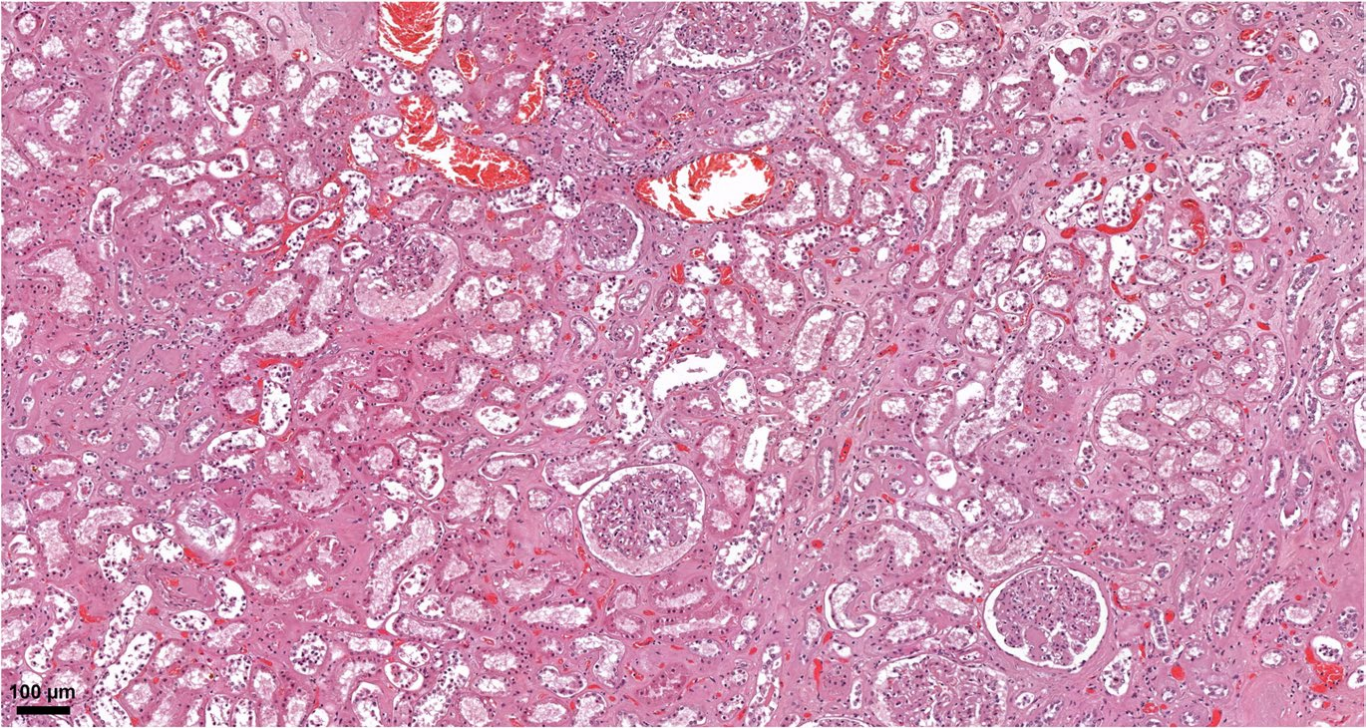
Histopathologic findings, kidney, in COVID-19. Extensive acute tubular necrosis, with tubular epithelial cell flattening, simplification, and sloughing. Peritubular capillaries are congested. Mild interstitial fibrosis can also be observed. No significant pathologic alterations in the glomeruli can be seen. HE.

The weight of kidneys was left 182 g (range: 50–360 g), right: 171 g (range: 58–360 g). The dominant histological alterations were chronic non-specific changes of the tubulointerstitium and moderate interstitial fibrosis/tubular atrophy (Figs. 4 and 11). In regard to the arteries, 51 specimens displayed at least 25 % narrowing of the arterial luminal diameter due to intimal fibroplasia. In 11 cases, histological correlates of end-stage renal disease could be established (diffuse global glomerulosclerosis, severe interstitial fibrosis/tubular atrophy). Changes characteristic of diabetes mellitus were present in 13 cases. In few samples, widespread fibrinoid necrosis was seen in the arcuate and interlobular arteries. In addition, hemorrhages in the parenchyma could also be seen. While glomerular changes were limited (only a couple glomeruli showed rare fibrin thrombi), these lesions were consistent with acute thrombotic microangiopathy. No focal-segmental glomerulosclerosis, immune complex-mediated or pauci-immune glomerulonephritis, viral cytopathic changes, or acute tubulointerstitial nephritis were found.

#### Liver

**Fig.12:**
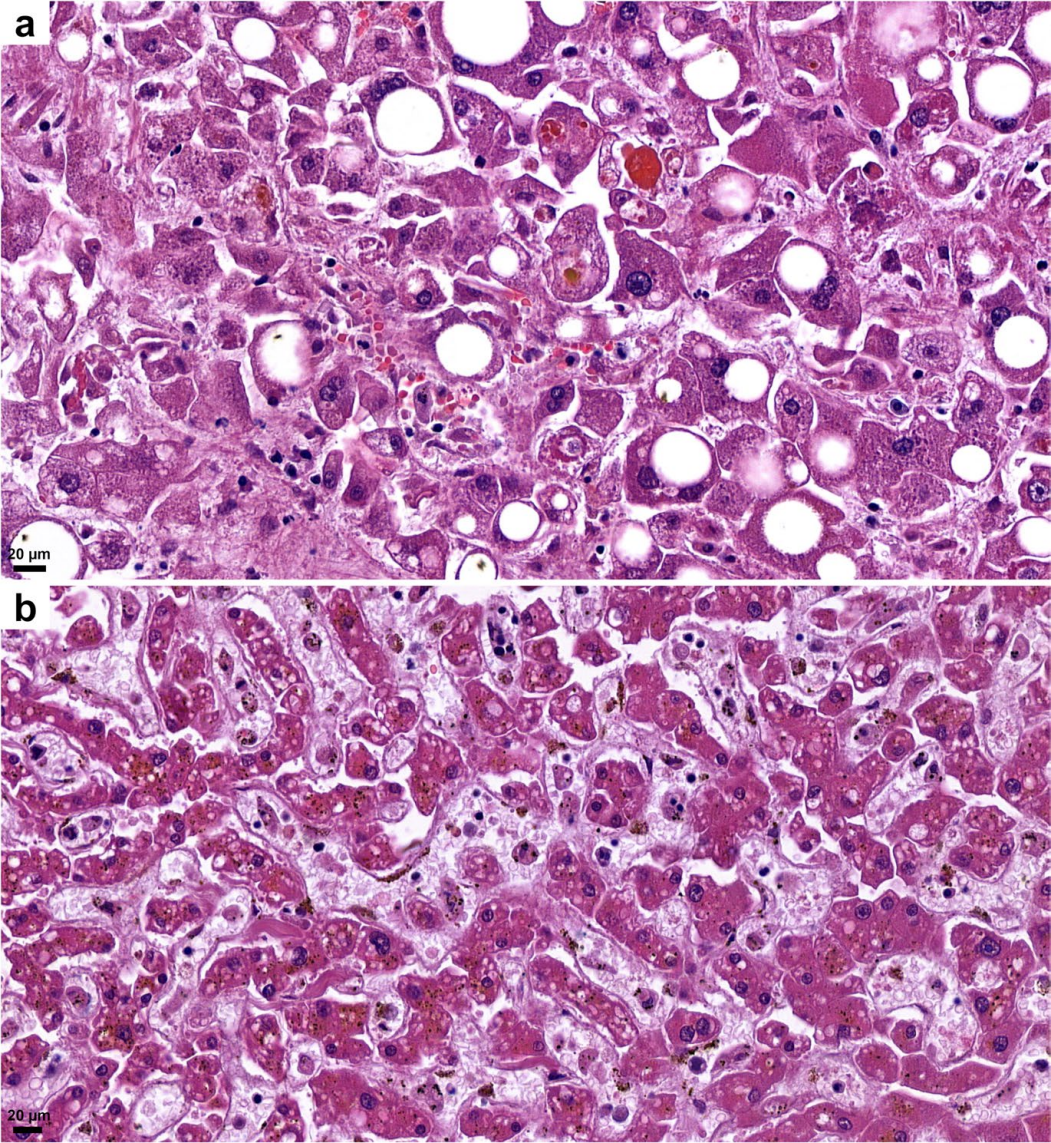
Histopathologic findings, liver, in COVID-19. Macro- and microvesicular steatosis, single cell hepatocyte death (apoptotic bodies, lytic necrosis) (**a**). Fibrin-like filamentous hematoxylinophilic material fills the dilated sinusoids, and sinusoidal endothelial damage (**b**). HE.

The weight of the liver on average was 1544 g (range: 520–3046 g). Vascular congestion was present in high number of samples (Fig. 5). Macro- and microvesicular steatosis were detected in 63 %, with varying grades (1–3) (Fig. 12a). No significant lymphocytic infiltration was observed; only very mild lymphocyte accumulation was seen in the portal areas. Mild portal fibrosis (F1–F2) was detected in 43 cases, while cirrhosis was seen in 4 cases. Single-cell hepatocyte death (apoptotic bodies and/or lytic necrosis) was often seen (Fig. 12a), without lobular inflammation. However, these findings could not be correlated with liver enzyme (ALT, AST) elevations. There was a more severe centrolobular zonal necrosis revealed in 11 cases. Fibrin-like, pale blue, occasionally fine filamentous material filled the dilated sinusoids, mainly in the centrolobular zone in several cases (Fig. 12b). The abruption of the central veins and severe damage of the vascular endothelium both in veins and sinusoids were seen (Fig. 12b). Cholestasis was detected in 52 cases; however, mainly mild and hepatocellular, more severe ductular cholestasis occurred only in 2 cases.

#### Central nervous system (CNS)

Cerebral specimens were subject to gross evalulation only in this series, because a detailed histo- and immunohistochemical analysis of the CNS is in progress and will serve as the basis for another study. Average weight of brains was 1250 g (range 1005–1590 g). The leading macroscopic alteration was the severe cerebral edema in 81 cases. Focal changes such as infarction were seen in 14 cases. Significant thinning of the cerebral cortex corresponding to cerebral atrophy was observed in 5 cases. Thrombotic vessel occlusion in small- and median-sized intracerebral vessels was not revealed macroscopically.

#### Other organs

No other significant alterations were detected in other organs, such as gastrointestinal tract, pancreas, and endocrine organs, which could be connected directly to COVID-19, except changes associated with pre-existing chronic diseases of the patients and thrombosis/thromboembolism in several small and large vessels.

### Categorization of cases reflecting the relevance of association with SARS-CoV-2 as cause of death

The direct underlying cause of death in COVID-19 in relation with the degree of association with SARS-CoV-2 infection was discussed and determined by the clinicians participating in the treatment of the patients and by the pathologists performing the autopsies and histopathological examinations of the deceased. The clinical data, co-morbidities, serum biochemistries, autopsy gross, and histology findings were presented and discussed on a case-by-case basis to reach a final conclusion. The definitions of the categories are summarized in Table 3.

**Table 3:** Definition of cause of death categories of deceased infected with SARS-CoV-2

Category	
Definition	
1st Category:	
Direct cause of death	Severe COVID-19 pneumonia: DAD, ARDS, pulmonary intravascular and intraalveolar fibrin coagulation
Strong association with COVID-19	Co-morbidities: not severe
2nd Category	
Direct cause of death	Severe pre-existing conditions
Contributive association with COVID-19	COVID-19 pneumonia as co-morbidity
3rd Category	
Direct cause of death	Other causes of death, unrelated to SARS-CoV-2 infection
Weak association with COVID-19	No COVID-19 pneumonia

Based on COVID-19 Guidelines for death certification and coding (WHO [30]), Edler et al. [10], *DAD* diffuse alveolar damage, *ARDS* acute respiratory distress syndrome

**Fig. 13:**
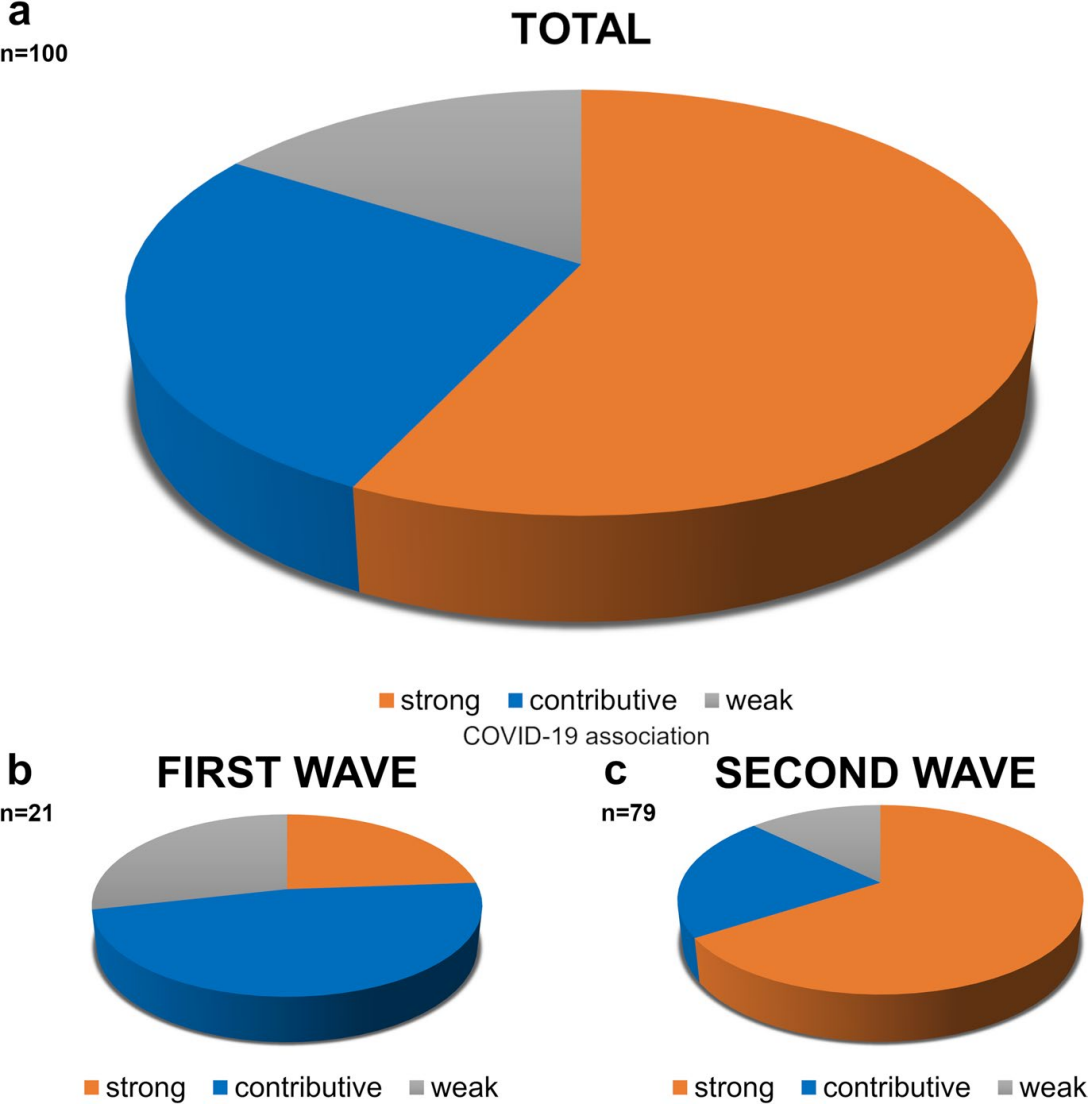
Numbers of subjects (total 100) in the three categories of “cause of death” association with SARS-CoV-2 infection, presented as pie charts. The largest number of subjects was in the strong COVID-19-association death category (57%), followed by the contributive (27%) and the weak (16%) association categories (**a**). The number of subjects in the first wave (between March and July 2020) was highest in the contributive COVID-19-association category (48%) and in the second wave (between August and December 2020) was highest in the strong association category (66%) (**b**)

Three categories leading to death were defined reflecting the relevance and contribution of COVID-19 infection. The majority of patients (*n*=57) were included in the *strong association with COVID-19 category (1)*, with cause of death directly due to SARS-CoV-2 infection and its sequelae. Another group of patients (*n*=27) classified to the *contributive association with COVID-19 category (2*) where death was caused by a pre-existing condition independent of COVID-19, yet the infection was not altogether irrelevant, therefore, contributing to death. A third cohort of decedents (*n*=16) belonged to the *weak association with COVID-19 category (3)*, in which viral infection had little or no effect on death. As the World Health Organization (WHO) defined “there is a clear alternative cause of death that cannot be related to COVID-19” (30) (Table 3 and Fig. 13a). There was a difference in the percentage of cases in the cause of death categories between the first wave, in which the contributive association with COVID-19 category cases were the most common and the second wave where the death with strong association with COVID-19 had the highest percentage (first wave, *n*, %; 24%, *48%*, 28 %, and second wave *n*, %; *66%*, 21%, 13%, *p*=0.002) (Fig. 13b, c).

Differences were seen among the cause of death groups in proportion of females [females, *n*, %; 24 (*42.1*), 14 (51.9), 12 (75.0); *p*=0.059] having the lowest proportion of females in the cohort with strong association with COVID-19 death category. The mean BMI [kg/m^2^; (SD); 30.41 (6.77), 26.77 (6.84), 27.33 (8.41); *p*=0.059] was the highest in death cases with strong association with COVID-19 (Suppl. Table 3).

Comparing co-morbidities, number of cases with the cardiovascular diseases (*p*=0.042) and tumors (*p*=0.003) were significantly higher among decedents with contributive association with COVID-19 category. Liver diseases, however, were higher among those with weak association with COVID-19 (*p*=0.025), similar to malignant tumors [*n*, %; 6 (10.5), 8 (29.6), 6 (*37.5*); *p*=0.015] (Suppl. Table 3). The highest proportion of patients who received intensive care belonged to the strong association with COVID-19 mortality category [*n*, %; 45 (*78.9*), 13 (48.1), 5 (31.2); *p*<0.001].

Percentages of deceased with elevated bilirubin, CKMB, LDH, and urea nitrogen values were significantly different in the cause of death categories. Elevated bilirubin (*p*=0.029) and elevated CKMB (0.018) were most frequently seen in the weak, while elevated LDH (*p*=0.047) and urea nitrogen (*p*=0.006) being the most common in cases with strong association with COVID-19 deaths (Table 2). Altered biochemistries were detected in high percentage (above 80% of the cases) of the different categories. Elevated CRP, D-Dimer, and interleukin-6 were seen in all three, high ferritin levels in both the strong and the weak, and low lymphocyte counts in the strong and the contributive, whereas high NT-proBNP levels in the contributive and the strong COVID-19-association mortality categories. GGT, LDH, and urea nitrogen were elevated only in fatal cases with strong association with COVID-19 category (Table 2).

**Fig. 14:**
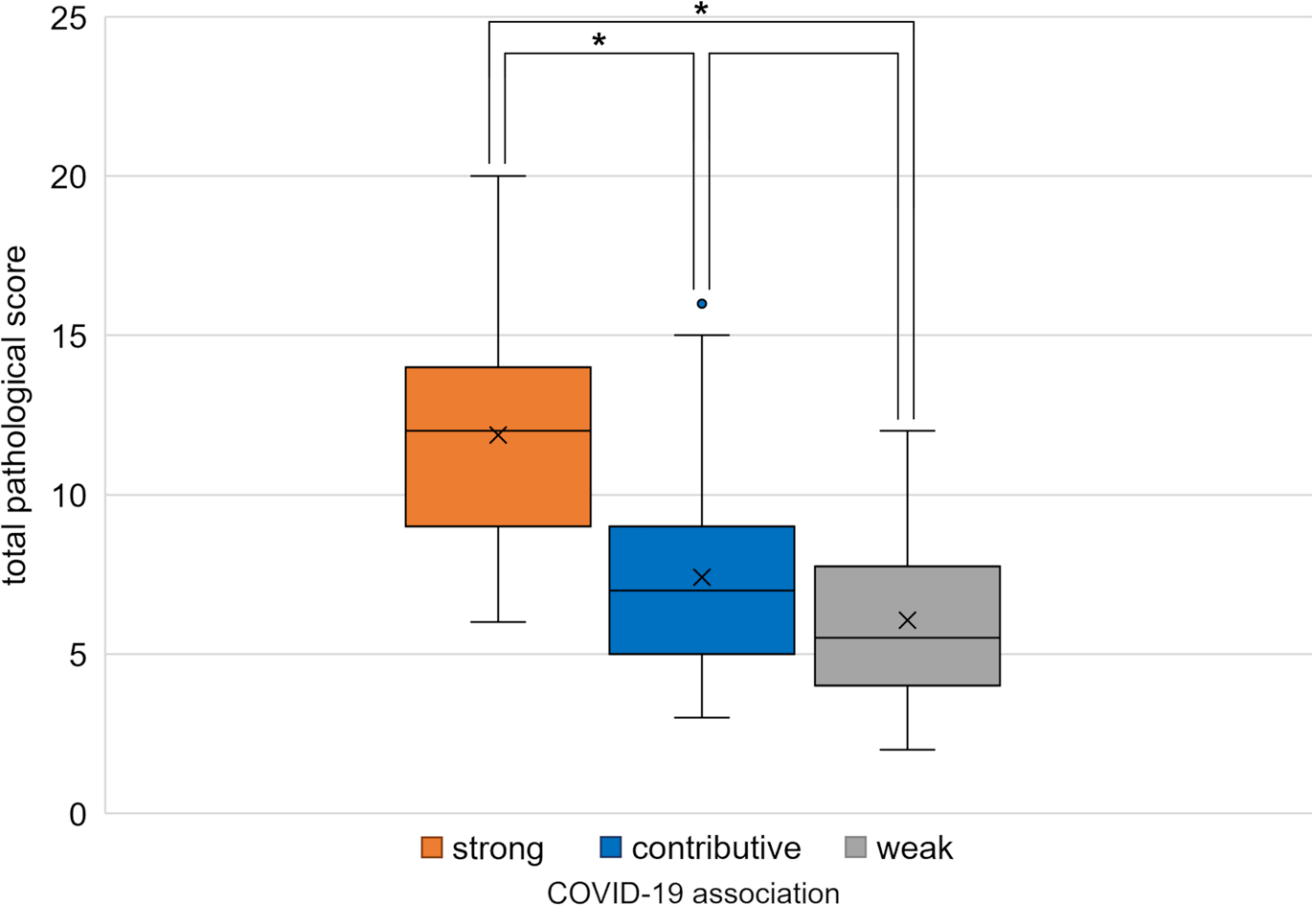
Comparison of the total scores of lung histopathologic findings, expressing the severity of the alterations in the three “cause of death” categories. Significant differences were found between the strong and contributive (*p*<0.0001) as well as between the strong and weak COVID-19- association categories (*p*<0.0001).

The histology severity scores (0–3) resulted in a complex evaluation score of different lung alterations, each case receiving a “total pulmonary histo-score” thereby summing up the severity of the individual alterations, such as fibrin microthrombi, hyaline membrane, fibrotic phase of DAD, alveolar edema, alveolar bleedings, pneumocyte hyperplasia, squamous cell metaplasia, alveolar macrophages, and lymphocytic infiltration. Adding the total lung histo-scores according to “the cause of death categories,” a highly significant difference was found between the strong and contributive, as well as between the strong and the weak association with COVID-19 fatal outcomes categories (*p*<0.0001). No significant difference was, however, detected between the death groups with contributive and weak association with COVID-19 (*p*>0.05) (Fig. 14). There was no change among the categories in the vascular congestion, neutrophil infiltration (bacterial superinfection), and pre-existing COPD (chronic obstructive pulmonary disease).

### Immunohistochemistry

**Fig. 15:**
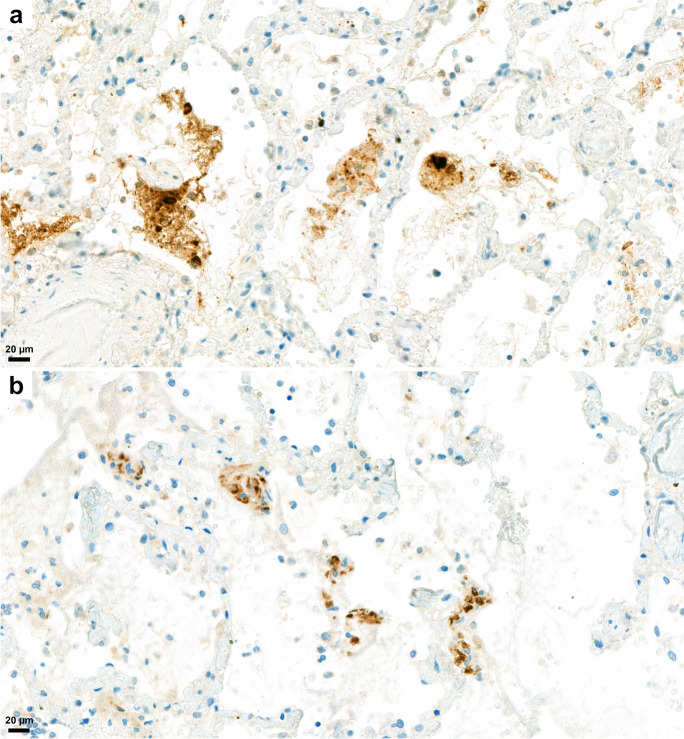
Immunohistochemical detection of SARS-CoV-2 virus in lung tissue. Brown marking for SARS-CoV-2 nucleocapsid protein is strong in cytoplasm and /or nuclei of detached cells, and as well as in debris, in the alveolar spaces (**a**). Brown marking for SARS-CoV-2 spike protein is detectable in alveolar walls and in pneumocytes (**b**). Antibody against nucleocapsid (**a**) and spike (**b**) proteins with diaminobenzidine chromogen and hematoxylin counterstain.

For detection of SARS-CoV-2 virus nucleocapsid and spike protein, immunohistochemical reactions were performed on lung tissue sections. Both nucleocapsid and spike viral proteins were detected by IH in the 25 cases in which autopsies were carried out <4 days PMI, and in 5–5 lung tissue slides taken 5–6, 7–8, and 8–10 days PMI. A patchy pattern of the viral protein distribution was observed. The nucleocapsid protein showed cytoplasmic and/or nuclear expression, whereas the spike protein showed only cytoplasmic expression in the pneumocytes. The intensity of the nucleocapsid protein immunostaining was generally higher than the spike protein, and both proteins were detected in significant amounts in detached cells or tissue debris present in intraalveolar localization (Fig. 15).

## Discussion

The 100 autopsy examinations analyzed here were performed from March through December 2020 by a single team in a single center, where autopsies are regularly done (approx. 2000 cases per year). The patients with COVID-19 were diagnosed and treated where they died, in the largest infectious-disease hospital in Hungary (Central Hospital of Southern Pest, National Institute of Hematology and Infectious Diseases, Budapest, Hungary, DPCK). SARS-CoV-2 sequences were demonstrated by RT-PCR testing in 3,814 patients during the study period, 495 died, and among them, 100 autopsies were performed in the Department of Forensic and Insurance Medicine, in collaboration with the 2^nd^ Department of Pathology of the Semmelweis University, Budapest, Hungary. Whether autopsy was conducted depended on random factors (number of autopsies to be performed, an availability of autopsy facilities and staff), as described for Graz, Austria [14]. To reduce interobserver variation in interpretation, with better consensus, images of HE-stained sections of tissue samples obtained at autopsy were scanned using a 3DHistech Pannoramic^®^ 1000 Digital Slide Scanner (Budapest, Hungary) and were discussed by the pathologists, an approach demonstrated to increase the value of histological evaluation by light microscopy [31].

During the pandemic of SARS-CoV-19 infection, several groups have described or reviewed the multiple organ alterations detected by autopsy examination alone [3–5, 8, 12–15, 17–24], or by autopsy supplemented with computer-tomographic examination [32]. Most of these publications present only a few autopsy cases or summaries of cases obtained from several centers, excepting a study from Hamburg [10] and, most recently, one from the Mount Sinai Hospital in New York [7].

The mean age of those autopsied in our study was 74.73 years (50 males 71.96 years; 50 females, 77.50 years, *p*=0.034). Most were aged between 70 and 80 years (34%), followed by those between 81 and 90 (23%) and those between 61 and 70 (23%). These data track those from other autopsy studies, in which average age were 69 years [8], 70.4 years [6], 79 years [17], and 80.5 years [14]. Co-morbidities detected (hypertension, cardiovascular diseases, diabetes, cerebrovascular diseases, chronic respiratory diseases, malignant tumors, renal and liver diseases) in our series’ subjects also tracked those in other studies [6, 14]. This demographic data suggests that the pathology findings in our study more likely reflect the COVID-19-associated changes than any particular to Hungary, even including the overweight (34%) and obesity (36%) found in the study population.

Among biomarkers, abnormal values for CRP, D-Dimer (>80%), IL-6, ferritin, urea nitrogen, GGT, LDH, NT-proBN, and neutrophil count were detected in >60% of patients. This agrees with demonstrations of elevated values for CRP, D-Dimer, IL-6, and ferritin in COVID-19 patients [8, 19, 33]. Increased CRP values reportedly are characteristic in COVID-19 but not in other viral infections [34]. Lymphopenia suggested as a marker of prognosis [34] was detected in 74% of our subjects.

Among affected organs, pulmonary injury was the most severe among on both macroscopic and histologic examination, as reported in previous studies [4, 5, 8–10, 14, 15, 19, 22, 35]. The most pronounced major histological findings were the bilateral, but non-uniformly distributed exudative, proliferative, and fibrotic stages of diffuse alveolar damage (DAD), with microthrombi and macrothrombi, also as noted by others [6, 8, 14, 17, 19, 35]. Features included fibrin-rich alveolar fluid, multinucleated giant cells, and an abundance of alveolar macrophages previously described [19]. A general impression garnered was that changes otherwise separately present in lung disease, such as usual interstitial pneumonia, desquamative interstitial pneumonia, and organizing pneumonia, in Covid-pneumopathy were superimposed on one another. According to Székely et al. [19], most coagulation events were detected in the intraalveolar and not in the intra-vascular spaces. The histopathological picture of COVID-19 pulmonary findings was classified as epithelial, vascular, and fibrotic patterns of injury, reflecting the different stages in pathophysiological “timeline” of the disease. This opinion was based on 42 publications reporting 198 cases, emphasizing that the three stages might occur consecutively or simultaneously, as suggested by Polak et al. [23].

SARS-CoV-2 nucleocapsid and spike proteins were detected immunohistochemically in pneumocytes and in detached cells in the alveolar lumen; coughed-up alveolar contents thus might transmit infective virus. Others also have immunohistochemically detected viral proteins in a similar distribution [6, 18] as well as viral RNA using in situ hybridization [19]. Bronchial changes were mainly associated with intubation, with severe purulent bronchitis with bacterial colonies noted. Characteristic so-called “ring” or “corona” bronchi were occasionally seen, surrounded by blood-filled, dilated small vessels, a phenomenon not described before in COVID-19. This was found principally in association with extensive passive hyperemia in the vicinity of the hemorrhagic infarctions and of sites of bronchopneumonia. The presence of ring bronchi was not correlated, however, with intubation or with severity of the bronchopneumonia. The bronchi, or even alveoli, of a few subjects contained foreign material (vegetable matter, perhaps regurgitated), or filamentous material (possible derived from filters used for ventilation).

The subject’s hearts were overweight, with dilated cavities. The myocardium was characterized histologically by wavy fibers. Coronary artery disease of varying extent was seen together with interstitial fibrosis. This combination probably constitutes a high-risk factor for COVID-19 death, as has been suggested [2, 36, 37]. Chronic myocardial damage and vessel remodeling indicated histories of cardiovascular ischemic disease originating years before fatal SARS-CoV-2 infection. Patchy hyperacute myocardial damages were frequently encountered, as seen by others [38]. This was randomly distributed rather than in the subendocardial accentuation of coronary heart disease and was not associated with inflammatory reaction usual in fully developed acute myocardial infarction. We thus concluded that this microischemic pattern reflected perimortem microcirculatory or cytokine-induced myocyte damages in a multiorgan-failure setting. Another form of acute myocardial circulatory derangement was (non-extensive) fibrin microthrombosis in 8% of patients, which appeared much less common than those in some other publications [39, 40]. The presence of neutrophils in intramyocardial capillaries and in ventricular endocardium was considered as sign of sepsis. Others detected myocarditis at autopsy in COVID-19 [32, 38, 41], which was present in 3% of our cases, a figure as low as those published by others [42]. The association between SARS-CoV-2 infection and increased number of myocardial macrophages and lymphocytes led to the conclusion that a “small fraction of the patients” might have myocarditis [43]. That high mortality in COVID-19 in patients is associated with a background of cardiovascular disease has been suggested [44], based on a summary of several studies and taking into account the pathogenesis of age-related vascular diseases as found in aged patients by Ungvari et al. [45].

Why the outcome of COVID-19 is more severe in older patients is unclear. Several factors are probably involved [44, 46, 47] as morbidities that include hypertension, cardiovascular diseases, diabetes, obesity, and respiratory and renal disease, all more common in those of older age, occur in high prevalence among patients dying in association with COVID-19. Further, contributing factors might include the “waning immunosenescence and age-related low level proinflammatory responses” some authors invoke [48], reducing the host’s innate immune response to infectious agents [46, 49]. Decline and dysregulation of immune function probably increase the susceptibility to a more severe COVID-19 clinical course in elderly people and simultaneously increase susceptibility to infection, involving age-related changes in both innate and adaptive immunity against viral infection [49]. Further, factors such as weaker Interferon type 1 response decline in de novo T cell response, unbalanced pro-inflammatory reactions leading to cytokine storm, etc. may conduce to more severe COVID-19 outcome and even death in the elderly patient [49]. ACE2, a receptor for SARS-coronavirus [48, 50], has a substantial protective role in cardiovascular functions [47, 51]. SARS-CoV-2 infection causes reduction in levels of ACE2 leading to inflammation and circulatory defects in several organs as in the lung, heart, kidney, liver, etc., leading to more severe COVID-19 [47].

No specific COVID-19-associated histopathologic alterations were detected in the kidney. Glomerulosclerosis, chronic non-specific changes of the tubulointerstitium, interstitial fibrosis, tubular atrophy, narrowing of the arterial lumina, diabetes-associated changes, thrombotic microangiopathy, and a few instances of end-stage renal disease were seen. Others reported mild acute tubular injury interpreted as suggesting reversibility [52] and focal segmental glomerulosclerosis [53].

Macro- and microvesicular steatosis of hepatocytes was present in 63% of subjects, as was a high rate of passive congestion (86%), as seen by others as well [6, 54, 55]. Mild hepatocellular cholestasis was seen in approximately half of subjects (52%), without a particular intralobular distribution, also previously reported [55]. Elevated bilirubin concentration and alkaline phosphatase activity were detected only on smaller proportions of subjects (respectively 15% and 28%). Diffuse, randomly distributed necrotic and apoptotic hepatocytes were seen often, but in small numbers. Centrilobular necrosis was present in 11 subjects, previously noted and suggested to be associated with hypoperfusion [6]. In addition, foci of multicellular hepatocyte necrosis were noted by others [20, 22]. Variable elevated AST and ALT activities were respectively found in 41% and 27% of subjects, as was variably elevated GGT activity (63%), as others also observed [36, 55, 56]. Asselah et al. [27] observed elevated biomarkers of hepatobiliary injury in 5 to 50 % of patients with COVID-19 in a pattern suggesting that liver disease was more likely hepatocellular than cholestatic, which is in agreement with our findings. Lymphocytic portal infiltration, albeit very mild, was seen in 47 subjects, together with mild portal fibrosis (F1–F2), and, in 4 of the 47, with cirrhosis. Others also have seen such inflammation [55]. No histological features of acute hepatitis with lobular inflammation were found, although others have noted mild changes of this sort in as many as 50% of those dying with COVID-19 [55]. We interpret our observation as suggesting that liver injury and dysfunction likely reflected secondary or toxic liver damage owing to drugs used in treatment, sepsis, and hypoxia/ischemia, viz. were multifactorial, rather than reflecting a direct viral cytotoxic effect here we concur with others [27].

Several categorizations of cause of death in the SARS-CoV-2-infected have discussed death “from” or “with” COVID-19 [18]. The 4 categories of definite, probable, possible, and not associated were applied in a single-center autopsy study from Hamburg [10]. We classed death in our 100 patients as strongly, contributively, or weakly associated with COVID-19. When association with SARS-CoV-2 infection was strong, death could be explained by alterations caused by COVID-19 with co-morbidities and organ alterations other than in the lung, insufficient to account for death (Hamburg “definite” category). When severe chronic disease antedated SARS-CoV-2 infection but would not in and of itself soon have led to death without the impetus of COVID-19, association of death with COVID-19 was considered contributive (Hamburg “probable” and “possible”). Assignments to this category were often controversial, prompting substantial disagreement and prolonged clinicopathologic discussions. The third category of weak association of death with COVID-19 included those, with severe pre-existing conditions likely soon to have led to death without viral infection (Hamburg “not-associated”).

Pathologists and clinicians discussed the autopsy findings, including histologic observations, at clinicopathological conferences where unanimity of interpretation was attempted. As presented above, most subjects exhibited strong (57) or contributive (27) associations between death and COVID-19 (84%). These results track those from Hamburg [10]. Cases were differently distributed among death-association categories in the first and second waves of pandemic. Contributive associations with COVID-19 were a plurality in the first wave (48%), followed by the weak (28%) and the strong (24%) categories. In the second wave, however, the strong association with COVID-19 category was in the majority (66%), followed by the contributive (21%) and weak (13%) categories. Death supervened significantly sooner after hospital admission in the second wave [mean survival time, days (SD): 12.26 (7.0)] than in the first wave [days (SD): 26.25 (22.27)], respectively. Survival with substantial pre-existent disease complicated by COVID-19 was substantially more likely, that is, in the second wave than in the first. The differences in clinical care that might underlie this improvement in survival remain to be ascertained.

That the hospital stay in the second wave of the pandemic was significantly shorter than that in the first might be explained by the finding that the percentage of deaths strongly associated with COVID-19 was significantly higher in the second than in the first wave. This is a counterpart, we believe, to the observation that lung alterations (as assessed by “lung histo-scores”) were significantly more severe in patients in the strongly COVID-19-associated death category than in patients in the contributively associated and weakly associated categories (*p*<0.0001). We consider these data to mean that deaths were predominantly due to pneumopathy. Patients with less severe lung disease, however (those in the contributively associated and weakly associated categories), survived longer, succumbing to co-morbidities whose treatment, although in the last analysis unfortunately not life-saving, prolonged their hospital stays.

In the first wave and in the second, patients with severe pneumopathy died. In the first wave but not in the second, patients with milder pneumopathy died. That is, by the time of the second wave, clinical care for COVID-19 was capable of rescuing from death a larger proportion of patients with relatively mild pneumopathy. Contributing factors might be greater alertness to the disorder, with speedier referral for care in less advanced stages of diseases, or better antivirals, or more expertise in ventilator management.

In conclusion, autopsies helped clinical caregivers understand to what extent the SARS-CoV-2 infection was responsible for the death of their patients and clarified both the pathological alterations that underlay clinical symptoms and the severity of organ alterations that led to death. Extensive lung injury was the principal cause of death in most subjects, reducing ventilatory capacity substantially via inflammatory infiltration, edema, and diffuse alveolar damage in its different phases. The thromboembolisms, both macro- and microvascular, caused the most pronounced alterations in several organs, particularly in the lung, followed by the heart, kidney, and liver. Analysis of the co-morbidities and of direct signs of viral infection, performed by clinicians and pathologists, permitted categorization of causes of death as strong, contributive, or weak association with COVID-19.

## Supplementary Information

Below is the link to the electronic supplementary material.

Supplementary file1 (DOCX 24 KB)
