# Supplementary material for: A COVID-19-association-dependent categorization of death causes in 100 autopsy cases
Source: GeroScience. 2021 Sep 11;43(5):2265–87. doi: 10.1007/s11357-021-00451-w (PMC8435112; doi:10.1007/s11357-021-00451-w)
Supplement: Supplementary file 1 — Supplementary file1 (DOCX 24 KB) [file 11357_2021_451_MOESM1_ESM.docx]

**Supplementary Table 1.**

The post-mortem interval (PMI) to autopsies varied between <1 to 10 days.

| Time from death to autopsy [day] | Number of cases |
| --- | --- |
| <1 | 7 |
| 1 | 16 |
| 2 | 9 |
| 3 | 11 |
| 4 | 15 |
| 5 | 8 |
| 6 | 10 |
| 7 | 9 |
| 8 | 4 |
| 9 | 7 |
| 10 | 4 |

**Supplementary Table 2.**

Demographic data and comorbidities in the different waves of deceased infected with SARS‑CoV‑2

| Patient characteristics | Pandemic waves | | p-value |
| --- | --- | --- | --- |
|  | first wave | second wave |  |
| n | 21 | 79 |  |
| Age, years; mean (SD) | 77.90 (14.33) | 73.89 (12.73) | 0.214 |
| Age categories, years; n (%) |  |  | 0.163 |
| <51 | 2 ( 9.5) | 5 ( 6.3) |  |
| 51-60 | 0 ( 0.0) | 3 ( 3.8) |  |
| 61-70 | 1 ( 4.8) | 22 (27.8) |  |
| 71-80 | 10 (47.6) | 24 (30.4) |  |
| 81-90 | 5 (23.8) | 18 (22.8) |  |
| >90 | 3 (14.3) | 7 ( 8.9) |  |
| Females; n (%) | 14 (66.7) | 36 (45.6) | 0.140 |
| Comorbidities in patient history; n (%) |  |  |  |
| Hypertension | 19 (90.5) | 66 (83.5) | 0.731 |
| Cardiovascular diseases | 14 (66.7) | 57 (72.2) | 0.601 |
| Diabetes | 9 (42.9) | 31 (39.2) | 0.805 |
| Cerebrovascular diseases | 7 (33.3) | 24 (30.4) | 0.796 |
| Respiratory diseases | 4 (19.0) | 26 (32.9) | 0.288 |
| Malignant tumors | 10 (47.6) | 10 (12.7) | 0.001 |
| Renal diseases | 5 (23.8) | 14 (17.7) | 0.539 |
| Diseases of the central nervous system | 6 (28.6) | 9 (11.4) | 0.080 |
| Liver diseases | 3 (14.3) | 3 ( 3.8) | 0.105 |
| BMI, kg/m2; mean (SD) | 27.83 (9.50) | 29.23 (6.50) | 0.433 |
| Length of hospital stay, days; mean (SD) | 31.86 (29.11) | 15.10 (18.41) | 0.002 |
| Length of hospital stay, days; median (IQR) | 24.00  [15.00, 38.00] | 10.00  [5.00, 17.50] | <0.001 |
| Length of hospital stay categories, days; n (%) |  |  | 0.056 |
| <1 | 0 ( 0.0) | 0 ( 0.0) |  |
| 1-2 | 1 ( 4.8) | 12 (15.2) |  |
| 3-4 | 0 ( 0.0) | 6 ( 7.6) |  |
| 5-9 | 2 ( 9.5) | 20 (25.3) |  |
| 10-15 | 4 (19.0) | 16 (20.3) |  |
| >15 | 14 (66.7) | 25 (31.6) |  |
| Intensive care = yes; n (%) | 10 (47.6) | 53 (67.1) | 0.129 |

**Supplementary Table 3.**

Demographic data and comorbidities in the different mortality categories of deceased infected with SARS‑CoV‑2

|  | Cause of death  association with COVID-19 | | | p-value |
| --- | --- | --- | --- | --- |
|  | strong  n (%) | contributive  n (%) | weak  n (%) |  |
| n | 57 | 27 | 16 |  |
| Age, years; mean (SD) | 74.58 (11.82) | 74.96 (16.41) | 74.88 (12.09) | 0.991 |
| Age categories, years; n (%) |  |  |  | 0.697 |
| <51 | 3 ( 5.3) | 3 (11.1) | 1 ( 6.2) |  |
| 51-60 | 2 ( 3.5) | 0 ( 0.0) | 1 ( 6.2) |  |
| 61-70 | 14 (24.6) | 7 (25.9) | 2 (12.5) |  |
| 71-80 | 20 (35.1) | 7 (25.9) | 7 (43.8) |  |
| 81-90 | 14 (24.6) | 5 (18.5) | 4 (25.0) |  |
| >90 | 4 ( 7.0) | 5 (18.5) | 1 ( 6.2) |  |
| Females; n (%) | 24 (42.1) | 14 (51.9) | 12 (75.0) | 0.059 |
| Comorbidities in patient history; n (%) |  |  |  |  |
| Hypertension | 49 (86.0) | 23 (85.2) | 13 (81.2) | 0.925 |
| Cardiovascular diseases | 40 (70.2) | 20 (74.1) | 11 (68.8) | 0.909 |
| Diabetes | 26 (45.6) | 9 (33.3) | 5 (31.2) | 0.456 |
| Cerebrovascular diseases | 17 (29.8) | 8 (29.6) | 6 (37.5) | 0.833 |
| Respiratory diseases | 17 (29.8) | 9 (33.3) | 4 (25.0) | 0.870 |
| Malignant tumors | 6 (10.5) | 8 (29.6) | 6 (37.5) | 0.015 |
| Renal diseases | 9 (15.8) | 6 (22.2) | 4 (25.0) | 0.598 |
| Diseases of the central nervous system | 4 ( 7.0) | 8 (29.6) | 3 (18.8) | 0.018 |
| Liver diseases | 2 ( 3.5) | 1 ( 3.7) | 3 (18.8) | 0.088 |
| BMI, kg/m2; mean (SD) | 30.41 (6.77) | 26.77 (6.84) | 27.33 (8.41) | 0.059 |
| Length of hospital stay, days; mean (SD) | 18.12 (21.06) | 18.19 (21.21) | 21.12 (27.56) | 0.886 |
| Length of hospital stay, days; median (IQR) | 14.00  [7.00, 20.00] | 12.00  [4.50, 21.50] | 15.50  [4.50, 26.50] | 0.775 |
| Length of hospital stay categories, days; n (%) |  |  |  | 0.650 |
| <1 | 0 ( 0.0) | 0 ( 0.0) | 0 ( 0.0) |  |
| 1-2 | 6 (10.5) | 4 (14.8) | 3 (18.8) |  |
| 3-4 | 2 ( 3.5) | 3 (11.1) | 1 ( 6.2) |  |
| 5-9 | 13 (22.8) | 6 (22.2) | 3 (18.8) |  |
| 10-15 | 13 (22.8) | 6 (22.2) | 1 ( 6.2) |  |
| >15 | 23 (40.4) | 8 (29.6) | 8 (50.0) |  |
| Intensive care = yes; n (%) | 45 (78.9) | 13 (48.1) | 5 (31.2) | <0.001 |
| Pandemic waves; n (%) |  |  |  |  |
| first wave | 5 ( 8.8) | 10 (37.0) | 6 (37.5) |  |
| second wave | 52 (91.2) | 17 (63.0) | 10 (62.5) |  |
